# Examining Canadian Smart Home Technology Users’ Perceptions of Privacy, Risks, Safety, and Data Collection Purposes: Cross-Sectional National Survey

**DOI:** 10.2196/70638

**Published:** 2026-09-23

**Authors:** Eva V Di Nallo, Charlene H Chu, Zara Hasan, Pratik K Mishra, Jacob Buchan, Tun Zheng, Andria Bianchi, Jesse Hoey, Shehroz S Khan

**Affiliations:** 1 Lawrence Bloomberg Faculty of Nursing, University of Toronto, 155 College St, Toronto, ON, Canada, 1 416-946-0217; 2 Institute for Life Course and Aging, University of Toronto, Toronto, ON, Canada; 3 KITE Research Institute, Toronto Rehabilitation Institute, University Health Network, Toronto, ON, Canada; 4 Rehabilitation Sciences Institute, University of Toronto, Toronto, ON, Canada; 5 Faculty of Health Sciences, McMaster University, Hamilton, ON, Canada; 6 Institute of Biomedical Engineering, University of Toronto, Toronto, ON, Canada; 7 Surrey Place, Toronto, ON, Canada; 8 Dalla Lana School of Public Health, University of Toronto, Toronto, ON, Canada; 9 David R. Cheriton School of Computer Science, University of Waterloo, Waterloo, ON, Canada; 10 College of Engineering and Technology, American University of the Middle East, Egaila, 54200, Kuwait

**Keywords:** smart home technology, user perspective, survey, cross-sectional study, privacy, purpose, safety, risk

## Abstract

**Background:**

Smart home technologies (SHTs) encompass internet-connected interfaces, sensors, monitors, devices, and appliances, which are networked together to allow for automation. They can facilitate tasks, such as taking medication or issuing fall alerts, and can control the home environment, such as by automating lighting. Their widespread use comes with concerns about power imbalances between users, technology companies, marketers, and state actors regarding data collection, its use and disclosure, and security issues, such as the potential for data breaches. SHT acceptance and adoption are shaped by user attitudes and trust, so understanding these factors is critical. A scoping review conducted by several authors of this paper explored user perceptions of SHTs and identified 4 key themes related to user attitudes: privacy concerns, knowledge of data collection, perceived risks and benefits, and perceived safety. Of the included studies in the review, none explored Canadian SHT users’ attitudes. Thus, little is known about how Canadians perceive and understand the privacy, data collection, and safety elements of their SHTs. Given Canada’s unique demographic diversity and distinct context, examining Canadian perspectives is necessary for advancing this body of research.

**Objective:**

This study explored users’ perceptions of SHTs in Canada, with a focus on privacy, the purpose of data collection, risks, and safety.

**Methods:**

An online survey was distributed across Canada between March 7 and April 24, 2023, to collect self-reported demographic information and users’ perceptions of SHT privacy, the purpose of data collection, risks and benefits, and safety associated with SHT use. To be eligible for this study, participants had to (1) be adults aged 18 years or older; (2) live in the community; (3) reside in Canada; (4) use at least one SHT; and (5) be comfortable communicating in English. The quantitative data were exported to SPSS (IBM Corp) and the Python programming language for descriptive analysis.

**Results:**

Self-reported survey data from a total of 881 SHT users were analyzed. Participants reported user mistrust (294/881, 33.4%), uncertainty (281/881, 31.9%), powerlessness (325/881, 36.9%), and digital resignation (232/881, 26.3%) pertaining to their SHT use. Many users displayed a willingness to trade privacy for perceived benefits, such as convenience, and some highlighted safety as a beneficial feature of SHTs. Contrary to previous internet or smart home research, most participants reported having read their SHT terms of use documents during setup.

**Conclusions:**

As the largest national survey of its kind in Canada, this study reveals a complex relationship between Canadians and SHTs, in which privacy concerns coexist with a desire for convenience and safety. These results highlight the need for greater transparency and trust-building measures in SHT design and policy. Understanding users’ perceptions is essential for developing technologies and user-centered frameworks that better align with their values and expectations in Canada’s diverse context.

## Introduction

### Background

Smart home technologies (SHTs) comprise devices that can personalize home services and facilitate everyday household tasks, such as automating lighting and controlling temperature. SHTs encompass internet-connected interfaces, sensors, monitors, devices, and appliances with embedded information technology systems, which are networked together to allow for automation and control of the home environment [1,2]. There has been a rise in the use of SHTs, with the penetration rate of SHTs in Canadian households expected to rise to nearly 60% by 2027 [3]. SHTs have been widely adopted by various individuals, including those of different ages, genders, and education levels [4]. Despite the growing prevalence of SHT use, considerations regarding the acceptability and adoption of these technologies are important, as research highlights that trust and risk awareness influence a person’s acceptability and adoption of SHTs [4]. Additionally, the increasing normalization of SHTs raises critical concerns about power imbalances between users, technology companies, marketers, and state actors regarding data collection, its use, and disclosure [5,6], as well as security issues such as potential data breaches [7]. The phenomenon of digital resignation, or the belief that privacy cannot be maintained despite user preferences, is prevalent among American SHT users due to the widespread culture of surveillance in everyday life [8,9]. This phenomenon is present in SHT users who have accepted a lack of privacy as a “necessary sacrifice” in return for the functionality and convenience supported by their technologies [10]. Additionally, scholars have theorized that privacy cynicism is a coping mechanism to reconcile the perceived inevitability of data breaches and exploitation with continued reliance on digital technologies, allowing users to rationalize their use of systems they distrust. Privacy cynicism encompasses attitudes such as uncertainty, powerlessness, and distrust [11]. Previous research from the United States has further illustrated that SHT users display limited knowledge of data usage practices by manufacturers and third parties [12,13].

Research highlights the important influences of acceptability attitudes, user perceptions, and trust on an individual’s adoption of SHTs, regardless of age, gender, and education level [4]. Building on existing literature, several of this study’s authors conducted a scoping review focusing on adults’ (aged 50 years or older) perceptions of SHTs, which identified 4 key themes related to user attitudes and concerns: privacy concerns, knowledge of data collection, perceived risks and benefits, and perceived safety [14]. This work highlighted important considerations, including privacy concerns related to corporate and third-party sharing, limited user knowledge of SHT data collection practices, perceived trade-offs between privacy and related values, and enhanced safety benefits [14]. Of the 15 studies in this review, none explored SHT user attitudes in Canada [14]. Additionally, only 1 study conducted during the pandemic explored barriers to SHT adoption in Canada, in which cost, privacy and security concerns, and unclear benefits of the devices were cited as barriers [15]. As Canada has unique demographic diversity and different legal frameworks for privacy at the federal and provincial levels, understanding attitudes governing Canadians’ use of SHTs is extremely important [16]. Below, we provide a summary of the 4 major themes (privacy perceptions, perceived purpose of data collection, risks and benefits, and safety) outlined in the review by Percy Campbell et al [14], supported by literature on adults aged 18 years and older generally, to provide context and motivation to conduct this survey exploring SHT user perceptions in Canada.

Privacy is a key aspect of SHT use, yet privacy policies for SHTs are often unclear or misunderstood [14]. Previous work highlights an overarching concern about the unclear manner in which third parties and service providers collect and handle user data [14]*,* as users have expressed concerns about negative implications following data sharing with third parties [17]. Research also shows that users’ perceived privacy and security within their homes are consistent with the strength of the relationships among users, manufacturers, and third parties [9]. If the relationships among these 3 groups are weak, users become concerned about compromised privacy and security [9]. The authors identified measures that can support the strengthening of these relationships, including government bodies issuing guidelines and regulations on privacy-protective measures [9]. Further, privacy concerns are related to the type of SHT used, as individuals perceive certain technologies, such as door sensors and health-monitoring technologies, as nonthreatening because they could support their independent living [18,19]. Therefore, it is important to consider the ways in which users of various SHTs perceive the privacy of their technologies.

Among SHT users of all ages, there are various perceptions of the purpose of data collection. A qualitative study exploring the perceptions of adults (aged 18+ years) regarding smart home purposes described that users believed data are analyzed by manufacturers for quality-improvement purposes and for the design of updated products [20]. Participants also noted that they believed devices, such as smart thermostats, were collecting information to support energy sustainability efforts [20]. Others believed that data were being collected to support marketing efforts, including targeted advertisements based on the users’ behaviors and market trends [20]. Older adult SHT users have expressed a limited understanding of why their data is being collected and how it is used, despite understanding the potential for privacy issues [21-24]. Specifically, Finnish older adults who use movement sensors expressed uncertainty regarding who had access to their data, whether they could access it themselves, and why it was being collected [23]. Across the literature, there is a trend that older adults have a limited understanding of the purposes for the collection of their SHT data [22-24].

Risks and benefits are a recurring theme in SHT user perception studies, as it is important for participants to weigh potential benefits and drawbacks when deciding whether to purchase or continue using SHTs [25]. A widely cited concern by users is related to data security threats [23,24,26-28], including fear of data breaches and user distrust in the SHT providers to adequately protect and use their information. Users have also cited concerns regarding exposure of financial information, profiling of their household activities by SHT providers, and the potential for safety consequences, as SHTs can detect when homes are not occupied, thereby increasing the risk of home invasions [24]. For older adult users, the literature indicates that their concerns relate to the malfunctioning of the technologies and affordability [14].

Despite the wide range of concerns related to SHT use, there is also a range of perceived benefits, including the opportunity for increased independence, reduced caregiver burden, and heightened daily confidence, especially for adults aged 50 years and older with functional challenges [23,27,29]. A study conducted in China found that perceived usefulness and ease of use of SHTs largely influenced one’s willingness to adopt and continue using SHTs [2]. Additionally, although SHT users express a wide range of perceived risks related to their use, some users believe the benefits outweigh the risks and accept them as a trade-off [20].

SHT users perceive a wide range of safety benefits related to their technologies. Notably, for users aged 50 years and older, there is a theme of excitement regarding their devices’ safety elements and their ability to support aging in place [19,23,26,29-32]. Specifically, SHT users in different geographic contexts, such as South Korea, the Netherlands, and the Philippines, have expressed enhanced feelings of safety related to their devices’ abilities to detect falls and share behavioral data with others, especially when retrieving their personal devices is challenging [19,29,30]. Additionally, Hjelm et al [33] found that smart homes can provide those in need of assisted living with more safety and agency by alerting caregivers if inconsistencies in behavior are displayed in the home, thereby reducing the need for in-home personal care [33]. Overall, across the literature, there is evidence of the safety-supporting benefits of SHTs as perceived by users.

Despite the exploration of SHT user perceptions in various geographical settings, to date, there is a lack of studies that focus on SHT users’ perceptions in Canada. Given Canada’s unique demographic diversity, examining Canadian perspectives is necessary to understand how these technologies are perceived, adopted, and integrated into daily life. Such research is essential for informing policies, design considerations, and strategies that address the specific needs, concerns, and expectations of Canadian users.

### Research Objectives

The aim of this study is to explore SHT users’ perspectives in Canada with regard to data protection attitudes and preferences. To achieve this aim, a survey was designed to gather insights into Canadian SHT users’ perceptions of privacy, the purpose of data collection, risks and benefits, and safety pertaining to SHT use, as these factors are deemed most relevant in the related literature [14].

This study addresses the following research questions:

1. What are SHT users’ attitudes toward privacy?
2. What are SHT users’ understandings of why and how their data is collected?
3. What do SHT users perceive about the possible benefits and potential risks and harms of SHTs?
4. What are SHT users’ perceptions about home safety in deploying these devices?

## Methods

### Study Design

This national study used a cross-sectional design. Individuals were invited to participate in an anonymous, 20-minute online survey designed around 4 major themes: privacy, purpose of data collection, risks and benefits, and safety. Questions were composed based on previous literature [14], and most were single-selection or multiple-choice questions, with an option to type a unique response. The questions were developed to understand the themes of privacy, purpose of data collection, risks, and safety. Questions aimed to understand whether users know where their data were stored and who has access to them, their perceptions of control over the collection and use of their data, the trade-off between the risks and benefits of their devices, and their feelings of safety in their homes with SHTs. Many survey questions used the Likert scale to measure statements of agreement, frequency, importance, quality, and likelihood related to the 4 themes mentioned above. The contents of the survey can be found in Multimedia Appendix 1.

The online survey was created using a secure, University of Toronto-hosted survey platform called REDCap (Vanderbilt University), in which both informed consent and data were obtained [34]. The survey was pretested with coapplicants on the grant and their graduate students to ensure question clarity before finalizing the questionnaire. The survey ran from March 7, 2023, to April 24, 2023. After completion of the survey, participants had the chance to provide their names to be included for a chance to win 1 of 8 gift cards to a coffee shop valued at CAD $25 (CAD $1=US $0.72 as of March 2023).

### Recruitment

Convenience and snowball sampling were used to recruit eligible participants across Canada for this study. A convenience sample was used, as the communication channel of our professional partners, namely the University of Toronto and AGE-WELL, was used to distribute information about the survey to recruit participants. Participants were also recruited by circulating information about the survey on social media platforms and research hosting services.

### Ethical Considerations

The survey was approved by the University of Toronto’s Research Ethics Board (protocol 43752). Informed consent was obtained using a secure, University of Toronto–hosted survey on REDCap [34]. Participants could withdraw from the study at any point prior to submitting the survey on REDCap. The data obtained from participants were deidentified and stored in password-protected files on the University of Toronto’s OneDrive and could only be accessed by authenticated members of the research team. All members of the team completed mandatory ethics training overseen by the project’s primary investigator.

### Inclusion and Exclusion Criteria

Eligible participants were defined as people who self-identify as 18 years or older, live in the community (ie, not in an institutional setting, such as long-term care or a group home), use at least one smart device, can communicate in English, reside in Canada, and can provide informed consent. Participants from all regions in Canada were eligible for this study.

### Statistical Analysis

Quantitative data were cleaned, downloaded from the survey platform in a Microsoft Excel format, and exported to SPSS and the Python programming language for analysis. Descriptive statistics, including frequencies and percentages, were used to describe sample characteristics and categorical data. The percentage values reported in the paper are rounded to the nearest integer for ease of reading.

## Results

### Data Cleaning

The survey yielded 1529 responses. Surveys that were incomplete (n=360), failed to provide consent (n=5), reported no ownership of a SHT (n=5), or had duplicate or invalid emails or phone numbers (n=278) were removed to ensure the quality of the final dataset. A survey was marked incomplete if REDCap detected that the survey was quit in the middle and was not submitted. For submitted surveys, an item that was left unanswered was denoted in the results with “no response.” The final dataset contained 881 survey responses.

### Sample Characteristics

Overall, a vast majority of respondents were under 40 years of age (821/881, 93.2%), 83.3% (734/881) were educated with at least a college or bachelor’s degree, and there was an almost equal distribution of males and females (Table 1). Detailed plots of the demographic survey data are available in Multimedia Appendix 2.

**Table 1.** Demographic table of survey respondents (N=881).

Demographic information	Frequency, n (%)
Age	
80-89	1 (0.1)
70‐79	4 (0.5)
60‐69	3 (0.3)
50‐59	12 (1.4)
40‐49	40 (4.5)
30‐39^a^	462 (52.4)
21‐29	228 (25.9)
18‐20	131 (14.9)
Sex	
Male^a^	447 (50.7)
Female	434 (49.3)
Ethnicity	
North American White^a^	666 (75.6)
African heritage	21 (2.4)
East Asian heritage	13 (1.5)
South Asian heritage	15 (1.7)
Prefer not to answer	4 (0.5)
Central American Indigenous heritage	17 (1.9)
North American Indigenous heritage	49 (5.6)
South East Asian heritage	2 (0.2)
South American Indigenous heritage	36 (4.1)
European heritage	33 (3.7)
Latin American	19 (2.2)
Middle Eastern heritage	5 (0.6)
Central Asian heritage	1 (0.1)
Education	
Bachelor’s degree^a^	369 (41.9)
College diploma	189 (21.5)
Graduate or professional degree	176 (19.9)
Apprenticeship or trades certificate	83 (9.4)
Secondary school (high school)	35 (3.9)
No formal schooling completed	22 (2.5)
Elementary school	5 (0.6)
Prefer not to answer	2 (0.2)
Income, (CAD $)	
100,000-125,000	83 (9.4)
75,000-100,000	209 (23.7)
50,000-75,000^a^	348 (39.5)
25,000-50,000	169 (19.2)
125,000-150,000	33 (3.7)
<25,000	22 (2.5)
>150,000	9 (1.0)
Prefer not to answer	8 (0.90)

a Values show the most prevalent group within each demographic category.

In terms of technology ownership, of the 881 participants, 572 (64.9%) reported using smart speakers (eg, Google Home, Amazon Alexa/Echo, and Google Nest/Google Nest Hub), 90 (10.2%) participants used security systems (eg, smart lock systems, Amazon Ring, and motion sensors), 32 (3.6%) reported using safety sensors (eg, fall detection sensors and emergency contact sensors), and 187 (21.2%) used other appliances (eg, smart lights, smart refrigerators, smart faucets, temperature sensors, humidity sensors, etc). Most participants owned their smart device for more than 2 years and reported being the primary user, meaning they used their SHT the most compared with other household members.

Table 2 provides a high-level overview of the results related to the 4 research questions. The proceeding results are organized according to the research questions and the category of SHT ownership.

**Table 2.** Perceptions of privacy concerns, data collection, potential risks and benefits, and safety features of smart home technologies (SHTs; N=881).

Category	Question and responses
Concerns over privacy	Question: Which of the following statements describes your concerns over privacy? Select all that apply. My private information could be collected and shared without my authorization (n=349, 39.6%)^a^ Companies can monitor me through my smart device (n=279, 31.7%) The government can monitor me (n=257, 29.2%) My smart device could be hacked (n=190, 21.6%) No concerns (n=164, 18.6%) Law enforcement can monitor me (n=157, 17.8%) Family members could spy on me with my smart device (n=64, 7.3%) My landlord could spy on me with my smart device (n=30, 3.4%) Roommates could spy on me with my smart device (n=16, 1.8%) Prefer not to answer (n=3, 0.3%) Family members can see me walking in with surprise birthday present! (n=1, 0.1%)
Data collected with SHT	Question: What kind of information do you think is collected? Select all that apply. I believe my smart device does not collect any personal information (n=599, 67.9%)^a^ Timing of use of my smart device (n=187, 21.2%) Location (n=113, 12.8%) Voice recordings (n=95, 10.8%) Search history (n=78, 8.9%) Video recordings (n=68, 7.7%) Motion (your movement within your home; n=51, 5.8%) No response (n=19, 2.2%) Not sure (n=9, 1.0%) Prefer not to answer (n=2, 0.2%) Layout of home (n=1, 0.1%) Sleep time (n=1, 0.1%)
Potential risks	Question: What potential risks were you concerned about? Select all that apply. I was concerned I could be monitored (n=290, 32.9%)^a^ I was concerned with losing my personal information (n=281, 31.9%) I was concerned my smart device would malfunction (n=275, 31.2%) I am not concerned about risks (n=263, 29.9%) I was concerned my smart device would underperform (n=204, 23.2%) I was concerned that my smart device could be hacked (n=191, 21.7%) Prefer not to answer (n=3, 0.3%)
Potential benefits	Question: What are the benefits of your SHT, if any? Select all that apply. Enhanced daily convenience (n=436, 49.5%)^a^ Enhanced safety (n=356, 40.4%) Low cost (n=325, 36.9%) Health benefits (n=287, 32.6%) Increased independence (n=227, 25.8%) Enhanced security (n=180, 20.4%) No benefits (n=20, 2.3%) Prefer not to answer (n=1, 0.1%) Entertainment (n=1, 0.1%)
Safety	Question: Does your SHT make you feel safer in your home? If yes, why do you feel safer? Yes. My smart device could detect and connect me to emergency services quickly if I am in danger (eg, fall, heart attack, and violence; n=347, 39.4%)^a^ Yes. I could connect with family (or vice versa) more easily during an emergency (n=316, 35.9%) I don’t feel safer (n=118, 13.4%) No response (n=105, 11.9%) Not sure (n=42, 4.8%) Prefer not to answer (n=5, 0.6%) Yes. Convenient and quick (n=1, 0.1%) Yes. Control over home environment (n=1, 0.1%) Yes. We have recorded evidence of any activity by those who should not be on our property (n=1, 0.1%)

a Values show the most prevalent response for each question.

### Privacy Attitudes

Participants’ attitudes toward privacy varied considerably across SHTs and are presented here by device type: smart speakers, security systems, safety sensors, and other connected appliances.

Among the 572 smart speaker users, 242 (42.3%) reported that their devices made their home feel less private. Despite this, the vast majority (n=522, 91.3%) reported adopting measures to protect their privacy, most commonly by avoiding sensitive conversations in the presence of their devices (n=268, 46.9%). However, many users (n=232, 40.6%) expressed uncertainty regarding the effectiveness of these precautions, acknowledging that ultimate control lay beyond their reach. A substantial proportion (n=482, 84.3%) indicated that they willingly sacrificed privacy for convenience, while nearly half (n=255, 44.6%) voiced concerns about companies monitoring them through their devices.

In contrast, of the 90 participants who used smart home security systems, 25 (27.8%) reported that the technology made their homes feel more private. Just under half (n=41, 45.6%) of security system users reported taking steps to safeguard privacy, typically through limiting privacy settings (27/41, 65.9%). Nonetheless, many felt their efforts could only provide partial protection (19/41, 46.3%). More than half (46/90, 51.1%) raised specific concerns that companies might collect or share their information without consent.

Among the 32 users of safety sensors, privacy concerns were framed differently. Many participants reported either that the device had no impact on household privacy (n=10, 31.3%) or that they did not take any measures to safeguard privacy (n=24, 75%). Two-thirds (n=21, 65.6%) reported sacrificing privacy for convenience, and three-quarters (n=24, 75%) expressed worry about family members being able to monitor them through these devices. Of the 8 (25%) who did take privacy-protecting actions, 5 (62.5%) acknowledged that these measures offered only limited protection.

Finally, among the 187 users of other connected appliances, privacy was rarely seen as a major concern. More than half (n=108, 57.8%) felt their devices did not affect the privacy of their homes, and 99 (52.9%) reported no specific concerns. Most (n=132, 70.6%) did not take any measures to safeguard their privacy, while only 25 (13.4%) believed they could protect it to some extent. Just under half (n=92, 49.2%) reported that they were not sacrificing privacy for convenience.

### Purpose of Data Collection

Among 572 smart speaker users, 485 (84.8%) reported that they did not believe their devices collected personal information. Of the 76 (13.3%) who believed their personal data were collected, 52 (68.4%) indicated that the time of their usage was tracked, and 47 (61.8%) believed that data collection was necessary for the function of the smart speaker. Almost all smart speaker users (548, 95.8%) reported that they had read their device’s terms and conditions.

Among 90 security system users, just over half (n=46, 51.1%) believed that information was collected only when the system was in use. Additionally, 37 (41.1%) reported that their time of use was tracked, and half (n=45, 50%) believed that personal information collection was essential for SHTs’ function. The majority of participants (n=76, 84.4%) indicated that they had read the terms and conditions for their security systems.

Among 32 safety sensor users, 12 (37.5%) believed their devices collected information when in use and 12 (37.5%) believed their devices regularly collected information but did not constantly monitor them. A total of 20 (62.5%) participants reported that their time of device use was tracked, and 14 (43.8%) believed that personal information was collected for their family to monitor. A total of 27 (84.4%) safety sensor users read the terms and conditions of their devices.

Of the 187 participants who used other smart appliances, 82 (43.9%) believed that no personal information was collected. Of the 102 (54.5%) who believed their personal information was collected, 78 (76.5%) believed their time of device use was tracked, and just over half (n=52, 50.9%) believed that information collection was necessary for the device to function. A total of 150 (80.2%) users read the terms and conditions of their smart appliance.

### Perceived Risks and Benefits

Among the 572 smart speaker users, 483 (84.4%) reported being concerned about device-related risks. Specifically, 245 (42.8%) expressed concerns over being monitored. The majority of participants (n=464, 81.1%) believed that the risks could be mitigated, and 427 (74.7%) indicated that they were less concerned about risks with extended use compared with when they first received their devices. Almost half of the participants (n=251, 43.9%) reported that they believed enhanced safety was a benefit of these devices, and the majority of participants (n=475, 83.0%) indicated that the benefits of the devices outweighed the risks.

Among 90 security system users, 53 (58.9%) reported having no concerns about risks. Of the 37 participants (41.1%) who reported concerns, 28 (75.7%) expressed concerns about the system malfunctioning, 23 (62.2%) believed that risks could be mitigated, and 18 (48.6%) reported being more concerned about continued device use than when they first received it. Among the 90 security system users, 66 (73.3%) identified daily convenience as a benefit, and 29 (32.2%) reported that the benefits of the device outweighed the risks.

Among the 32 safety sensor users, 17 (53.1%) reported no concerns about risks related to their device. Of the 15 (46.9%) who expressed concerns, 11 (73.3%) expressed concerns about their device malfunctioning, and 7 (46.7%) believed that risks could be mitigated. Of those 15 participants, 6 (40%) reported being less concerned than when they first received their device. Among 32 users, 22 (68.8%) identified enhanced safety as a benefit, and 13 (40.6%) reported that benefits outweighed risks.

For the 187 other smart appliance users, 105 (56.1%) reported no concerns about the risks. Of the 81 (43.3%) who expressed concerns, 34 (41.9%) flagged malfunction as a risk, 63 (77.8%) believed the risks could be mitigated, and 40 (49.4%) were more concerned about risks with extended use than when they first received the SHT. Of the 187 users, the majority (n=153, 81.8%) cited enhanced daily convenience as a top benefit of smart home appliances, and 69 (36.9%) users believed that the benefits of SHT use outweighed the risks.

### Safety Perceptions

Of the 572 smart speaker users, 502 (87.8%) indicated that they felt that smart speakers made them feel safer at home. Of those 502 participants, 286 (56.9%) indicated that they helped them connect with their families during emergencies.

Among the 90 security system users, 57 (63.3%) indicated that their device made them feel safer. Among those 57 users who indicated an increased perception of safety, 18 (31.6%) indicated this was due to the SHTs’ ability to detect emergencies and connect them with emergency services and their families.

Of the 32 safety sensor users, 28 (87.5%) indicated that their SHT made them feel safer in their homes. Of those 28 users, 12 (42.9%) indicated that this was because it helped them detect emergencies and connect them to emergency services when needed.

For the 187 other smart appliance users, 78 (41.7%) indicated that their SHT did not make them feel safer in their homes. However, of the 65 (34.8%) who indicated a higher perception of safety, 40 (61.5%) attributed this to their SHTs’ ability to connect them to emergency services when in danger.

## Discussion

### Principal Results

To our knowledge, this is the largest survey in Canada to explore SHT users’ perceptions of privacy, the purpose of data collection, risks and benefits, and safety. The results of this study can be used to target future research and policies related to SHTs. We discuss the findings below to address each of the 4 research questions.

### Privacy Attitudes

The survey findings highlight the presence of privacy cynicism among SHT users, as many felt that their privacy was being compromised but were not confident in their ability to mitigate it. Privacy cynicism results when users feel that they cannot prevent the loss of privacy, so they neglect engaging in protective behaviors [35]. Lutz et al [11] explored privacy cynicism with a group of 1008 survey respondents in Germany and found that it was often composed of 4 significant factors: mistrust, uncertainty, powerlessness, and resignation [11,35].

All 4 of the components of privacy cynicism are displayed within our smart speaker user sample. Specifically, of the 881 participants, 294 (33.4%) of users believed that SHTs made their homes less private, thereby compromising user trust (mistrust factor); 281 (31.9%) users were uncertain whether their efforts to safeguard their privacy would be effective (uncertainty factor); 325 (36.9%) users believed their privacy was ultimately out of their control (powerlessness factor); and 232 (26.3%) had concerns about being monitored via their devices and were uncertain whether they could protect their information (resignation factor). Despite being concerned about privacy breaches and admitting that smart speakers compromise privacy, users persisted in using SHTs as they believed they could not control this risk. Smart speaker users agreed that they were knowingly sacrificing privacy for convenience, highlighting the prevalence of privacy cynicism and digital resignation. Similarly, Lutz and Newlands [36] noted a general lack of privacy protection behaviors among smart speaker users in the United Kingdom, offering privacy cynicism as an explanation. By applying the framework outlined in Lutz et al [11] to SHTs, these phenomena may be handled by companies through designs and policies that restore user agency and an internal locus of control [11]. This can be accomplished in many ways, including opt-in options for user data collection through Privacy by Design principles [37] and embedded permission requests if certain actions may compromise personal data. Greater transparency in user manuals or through educational content on limiting device monitoring can also address privacy concerns. However, the onus of responsibility should primarily rest with legislators and regulators, as opposed to individual users.

Ultimately, the aforementioned behavioral patterns were not reflected to the same extent among security system users in this study. Security system users believed that privacy could be controlled to an extent, but not fully. Participants highlighted methods of privacy protection, such as disabling certain features, avoiding sensitive conversations around certain devices, unplugging SHTs when not in use, limiting privacy settings, and updating software. These mitigation strategies share similarities with those described in a previous qualitative study, which included limiting audio and video exposure, selecting SHTs with strong security features, and limiting the information they share with devices [38].

SHTs listed in the safety sensors category were not widely correlated with privacy concerns in our findings. This may be due to the extent to which safety sensors are related to health care or caregiving and users perceive strong access controls to sensitive health-related data [39]. Despite this, some users still agreed that their privacy was sacrificed, and some users limited device privacy settings. The literature highlights that for smart sensors to be effective, they require access to personal information [40], which might explain the low prevalence of privacy concerns. Our findings may further be explained by research that shows that privacy-concerned users are less likely to continue their use of smart sensors, highlighting that those who continued their use, such as the participants in this study, do not perceive a large risk [40].

Users of other smart appliances were the only group that did not believe they were sacrificing privacy for convenience. Devices in this category include smart fridges, smart lights, smart faucets, temperature sensors, and humidity sensors. Previous work aligns with this study’s findings, as there is a widespread belief that these devices do not present the same kinds of threats as other smart devices, despite them consisting of sensors that are able to collect and reveal personal data about users’ locations, identities, activities, usage patterns, and touch screen inputs [41].

As noted in a previous review, smart speaker users are often unaware of the types of information collected [14]. Many are uncomfortable sharing data with manufacturers, household members, or third parties [26]. These considerations may suggest that smart speaker users have the greatest privacy expectations and, subsequently, that this SHT group is a good area for policy and manufacturers to target when aiming to improve SHT user data protection. Reconsidering privacy legislation in Canada could also be beneficial for protecting SHT users. Current federal legislation and proposed reforms have been criticized for their emphasis on catering to business needs as opposed to recognizing privacy as a human right [42]. Quebec’s recent Law 25 more closely parallels the EU’s General Data Protection Regulation and should be followed by other Canadian jurisdictions [43,44].

### Purpose of Data Collection

The majority of participants, irrespective of the type of SHT, reported that they read the terms and conditions for their device in detail. This trend is unexpected since the literature has widely reported that internet users tend not to read the terms and conditions, especially in the context of social media [45]. As prior research suggests, terms of use agreements are frequently characterized by dense jargon and extensive length [46]. It is worth noting that this observation may be influenced by the demographic composition of the surveyed users, with approximately 42% (369/881) of respondents having attained at least a bachelor’s degree.

Many users of smart speakers and other appliances did not believe that their personal information was collected by their SHT. This is contrasted with users of security systems, who believe their device does collect some information when in use. This contrast is likely due to user perceptions of the information that devices need to function, such as access to personal information when in use or upon setup. A similar pattern of responses emerged with safety sensor users. These users mostly agreed that data were collected regularly (eg, time of use of SHT, voice recordings, search history, video recordings, motion within the home, etc). Again, these trends show some relation between perception of the purpose of SHT and understanding of data collection.

### Perceived Risks and Benefits

Smart speaker users were often concerned about the risk of being monitored. Many agreed, however, that enhanced safety was a benefit of their SHT and that the benefits of enhanced safety reasonably outweighed the risks of their SHT. This is in contrast with users of security systems, safety sensors, and other appliances, who were largely unconcerned about potential risks.

Notably, among security system users who expressed concerns about their devices, a significant number reported that their concerns intensified over time. In contrast, users of safety sensors experienced a decrease in their concerns over time. Additionally, participants consistently recognized improved safety as a primary benefit of smart speakers and safety sensors, while they associated enhanced convenience with security systems and other appliances. It is evident that, regardless of user group, participants agree that the benefits of their SHTs outweigh the risks, which could be their justification for continuing the use of their devices. These findings are consistent with previous literature on the topic in various geographic locations, including Europe and Asia [28,47-50], and could be related to users’ biases that SHTs are safe to use, overlooking other potential risk factors.

### Safety Perceptions

A prevalent theme among smart speaker, security system, and safety sensor users is that they felt safer with these SHTs due to the ability to quickly connect with external support, including family or emergency services, when they were incapable of doing so independently. Users of other appliances did not feel an enhanced sense of safety from SHT ownership. This may be because these appliances, such as humidity sensors, are marketed for enhancing quality of life within the home [51] and may not be widely understood to enhance personal safety.

Convenience was reported as a primary reason for adopting SHTs. The majority of survey respondents used smart speakers, which can be used to control other smart devices in the home (eg, Google Home, Amazon Alexa/Echo, and Google Nest/Google Nest Hub). The emphasis on convenience through the 4 research questions has also been confirmed by previous studies conducted in the United States [52-54], as well as a study by Dong et al [55] in China, which reported that convenience significantly impacted users’ perceptions of SHT usefulness and, subsequently, SHT use [55]. This is not unexpected, as SHTs are often advertised and sold with the promise of convenience [56]. This study highlights avenues through which policy around SHTs may change to further benefit users, including user-centric design by equipping SHTs with Privacy by Design principles and embedded permission requests, as well as greater transparency within user manuals and promotional content. Private-sector privacy law reform that reflects user concerns and experiences with SHTs would also be beneficial. This may be especially important for SHTs used for health purposes [14].

### Limitations

Several limitations may impact the generalizability of the findings reported in this study. Most importantly, some of our findings may be biased due to self-report by respondents. Additionally, including only current owners of SHTs may have skewed the results, as considering the perspectives of past and prospective SHT users would have provided a more comprehensive understanding of perceptions related to SHT privacy, data collection purposes, risks and benefits, and safety. Also, the majority of survey respondents were English speakers, highly educated, young (in their 30s), and high-income earners, which does not reflect the diversity of Canada’s population. As a result, this study’s participants may approach SHT use differently and share differing perspectives from SHT users of different backgrounds, such as those who are older and less educated. This is an important consideration, as the literature indicates that older adults may have different attitudes and experiences with technology than younger populations [57].

Another limitation is that despite the survey’s targeted distribution within Canada through team efforts and social media outreach, there remains the potential for participation by individuals residing outside of Canada, as answers were self-reported and there was no way for the research team to verify geographical location. Lastly, the survey questionnaire was administered in English, so individuals who are unable to read and write in English would not have participated, which limits our sample’s ability to reflect the diversity of Canada’s population.

### Future Work

The extent to which the perceived SHT purpose relates to user acceptance of data collection should be explored and disseminated in future studies. Future work should aim to recruit a more representative sample of Canada’s population to understand whether perceptions differ across various backgrounds. Future efforts should also consider how past, present, and prospective SHT users perceive the purposes of SHT data collection, the risks and benefits, and privacy and safety elements to further understand how these factors influence adoption and continued use of SHTs. Moreover, moving beyond cross-sectional snapshots of perceived privacy by adopting longitudinal and/or mixed methods approaches will enable an understanding of nuances influencing adoption, coadaptation between devices and users, and continued use of these technologies. This will support a broader understanding of important factors related to perceived privacy, safety, and risks and benefits that have large influences on the use of SHTs. Future work should also focus on creating regulatory reforms and adopting user-centric frameworks to ensure that SHTs align with the needs and preferences of users to optimize their benefits.

### Conclusions

This national survey, the largest of its kind in Canada, reveals a complex relationship between Canadians and SHTs, where privacy concerns coexist with a strong desire for convenience and safety. Canadian users frequently exhibit privacy cynicism in exchange for perceived benefits. Concerns about unauthorized data collection and surveillance by companies, the government, and other actors are prevalent, with many users feeling resigned to the inevitability of privacy loss, reflecting patterns of digital resignation. This study identifies gaps in user awareness about data collection, with many underestimating the extent of data gathered by their devices. Addressing these perceptions through user-centric frameworks and regulatory reforms is essential for aligning SHT development with the values and expectations of Canada’s diverse population.

## Supplementary material

10.2196/70638 Multimedia Appendix 1 Questions and responses from national survey.

10.2196/70638 Multimedia Appendix 2 Results of the smart home technology (SHT) survey presented to participants.

## Abbreviations

SHT: smart home technology
