# Supplementary material for: Examining Canadian Smart Home Technology Users’ Perceptions of Privacy, Risks, Safety, and Data Collection Purposes: Cross-Sectional National Survey
Source: JMIR Form Res. 2026 Sep 23;10:e70638. doi: 10.2196/70638 (PMC13600659; doi:10.2196/70638)
Supplement: Supplementary file 1 [file formative-v10-e70638-s001.pdf]

Demographic Information You may select "prefer not to answer" if you are uncomfortable or unsure of how to respond to any of the questions. All identifiable information identity will remain confidential.

What is your age, in years?	◯ 18-20<br>◯ 21-29<br>◯ 30-39<br>◯ 40-49<br>◯ 50-59<br>◯ 60-69<br>◯ 70-79<br>◯ 80-89<br>◯ 90+<br>◯ Prefer not to answer
What is your highest level of completed education?	◯ No formal schooling completed<br>◯ Elementary school<br>◯ Secondary school (High school)<br>◯ College diploma<br>◯ Apprenticeship or trades certificate<br>◯ Bachelor degree<br>◯ Graduate or professional degree<br>◯ Prefer not to answer
What is your annual income, in Canadian dollars (CAD)?	◯ Less than $25,000<br>◯ $25,000 to $50,000<br>◯ $50,000 to $75,000<br>◯ $75,000 to $100,000<br>◯ $100,000 to $125,000<br>◯ $125,000 to $150,000<br>◯ Over $150,000<br>◯ Prefer not to answer
What is your ethnicity?	◯ North American Indigenous heritage (First Nations, Metis, Innu...)<br>◯ Central American Indigenous heritage (Chorotegan, Lencan, Tawahkan...)<br>◯ South American Indigenous heritage (Atacaman, Cayubaban, Chimilan...)<br>◯ Latin American (Mexican, Venezuelan, Brazilian...)<br>◯ North American White<br>◯ European heritage (Western European, Northern European, Eastern European, Southern European, Central European...)<br>◯ African heritage (West African, East African, South African, North Africa, Central African...)<br>◯ East Asian heritage (Chinese, Japanese, Korean...)<br>◯ South Asian heritage (Indian, Sri Lankan, Nepalese...)<br>◯ South East Asian heritage (Indonesian, Filipino, Maori...)<br>◯ Central Asian heritage (Kazakh, Turkmen, Mongolian...)<br>◯ Middle Eastern heritage (Levantine, Persian, Yemeni...)<br>◯ Prefer not to answer
What is your gender identity?	◯ Male<br>◯ Female<br>◯ Other<br>◯ Prefer not to answer

(Optional): specify gender identity.

____

Smart Home Object

What smart home devices do you own? Select all that apply.

- ☐ Amazon Alexa smart speaker
- ☐ Amazon Ring
- ☐ Google Home
- ☐ Google Nest/Google Nest Hub
- ☐ Smart Lock System
- ☐ Smart Fridge
- ☐ Smart Lights
- ☐ Smart Faucet
- ☐ Temperature sensors
- ☐ Motion sensors
- ☐ Humidity sensors
- ☐ Fall detection sensors
- ☐ Emergency contact sensors
- ☐ Other
- ☐ I do not own any smart home object

Please specify "other":

____

Please select the smart home object you use most often. If you don't know which you use most often, select one object you use frequently.

The rest of the survey will focus on the object you select here.

- ◯ Amazon Alexa smart speaker
- ◯ Amazon Ring
- ◯ Google Home
- ◯ Google Nest/Google Nest Hub
- ◯ Smart Lock System
- ◯ Smart Fridge
- ◯ Smart Lights
- ◯ Smart Faucet
- ◯ Temperature sensors
- ◯ Motion sensors
- ◯ Humidity sensors
- ◯ Fall detection sensors
- ◯ Emergency contact sensors
- ◯ Other
- ◯ I do not own any smart home object

Please specify "Other":

____

Smart Home Object Information

Why did you purchase your [q1_shtobject]?	◯ I bought my [q1_shtobject] to help me improve my daily life<br>◯ I am interested in owning the latest technology<br>◯ The [q1_shtobject] can connect with other technology I own<br>◯ The [q1_shtobject] was on sale or was free from a promotion<br>◯ Someone from my support network (e.g., family, friend, caregiver) purchased the [q1_shtobject] for me<br>◯ Other (please specify)<br>◯ Prefer not to answer
Please specify "Other":	__________________________________
Why did you purchase your [q1shtobject_other]?	◯ I bought my [q1shtobject_other] to help me improve my daily life<br>◯ I am interested in owning the latest technology<br>◯ The [q1shtobject_other] can connect with other technology I own<br>◯ The [q1shtobject_other] was on sale or was free from a promotion<br>◯ Someone from my support network (e.g., family, friend, caregiver) purchased the [q1shtobject_other] for me<br>◯ Other (please specify)<br>◯ Prefer not to answer
Please specify "Other":	__________________________________
How long have you owned your [q1_shtobject]?	◯ Under 6 months<br>◯ Over 6 months<br>◯ Over 1 year<br>◯ Over 2 years<br>◯ Over 3 years<br>◯ Over 4 years<br>◯ Over 5 years<br>◯ Over 10 years<br>◯ Prefer not to answer
How long have you owned your [q1shtobject_other]?	◯ Under 6 months<br>◯ Over 6 months<br>◯ Over 1 year<br>◯ Over 2 years<br>◯ Over 3 years<br>◯ Over 4 years<br>◯ Over 5 years<br>◯ Over 10 years<br>◯ Prefer not to answer
Do you use your [q1_shtobject] more than anyone else who spends time in your home?	◯ Yes<br>◯ No<br>◯ Prefer not to answer
Do you use your [q1shtobject_other] more than anyone else who spends time in your home?	◯ Yes<br>◯ No<br>◯ Prefer not to answer

If not you, who uses it the most?	◯ Spouse/partner<br>◯ Child<br>◯ Parents/guardians<br>◯ Extended family member<br>◯ Landlord<br>◯ Caregiver<br>◯ Roommate<br>◯ Other (please specify)<br>◯ Prefer not to answer
Please specify "Other":	______________________________

Section 3: Privacy

The following questions will explore your opinion on privacy related to your [q1_shtobject].

The following questions will explore your opinion on privacy related to your [q1shtobject_other].

Select the option that best matches your feelings about privacy in regard to your [q1_shtobject].

- ◯ My [q1_shtobject] has not altered my feelings of privacy at home; nothing has changed
- ◯ My [q1_shtobject] makes my home seem less private
- ◯ My [q1_shtobject] has invaded my privacy, but so do my other objects and devices
- ◯ There is no privacy in society anyway, so my [q1_shtobject] makes no difference
- ◯ My [q1_shtobject] has increased my sense of privacy at home
- ◯ Other (please specify)
- ◯ Prefer not to answer

Please specify "Other":

____

Select the option that best matches your feelings about privacy in regard to your [q1shtobject_other].

- ◯ My [q1shtobject_other] has not altered my feelings of privacy at home; nothing has changed
- ◯ My [q1shtobject_other] makes my home seem less private
- ◯ My [q1shtobject_other] has invaded my privacy, but so do my other objects and devices
- ◯ There is no privacy in society anyway, so my [q1shtobject_other] makes no difference
- ◯ My My [q1shtobject_other] has increased my sense of privacy at home
- ◯ Other (please specify)
- ◯ Prefer not to answer

Please specify "Other":

____

Which of the following statements describes your concerns over privacy and your [q1_shtobject]? Select all that apply.

- ☐ Companies can monitor me through my [q1_shtobject]
- ☐ The government can monitor me
- ☐ Law enforcement can monitor me
- ☐ My private information could be collected and shared without my authorization
- ☐ My [q1_shtobject] could be hacked
- ☐ Family members could spy on me with my [q1_shtobject]
- ☐ Roommates could spy on me with my [q1_shtobject]
- ☐ My landlord could spy on me with my [q1_shtobject]
- ☐ No concerns
- ☐ Other (please specify)
- ☐ Prefer not to answer

Please specify "Other":

____

Which of the following statements describes your concerns over privacy and your [q1shtobject_other]? Select all that apply.	☐ Companies can monitor me through my [q1shtobject_other]<br>☐ The government can monitor me<br>☐ Law enforcement can monitor me<br>☐ My private information could be collected and shared without my authorization<br>☐ My [q1shtobject_other] could be hacked<br>☐ Family members could spy on me with my [q1shtobject_other]<br>☐ Roommates could spy on me with my [q1shtobject_other]<br>☐ My landlord could spy on me with my [q1shtobject_other]<br>☐ No concerns<br>☐ Other (please specify)<br>☐ Prefer not to answer
Please specify "Other":	________________________________
Do you take any measures around your [q1_shtobject] to protect your privacy at home?	◯ Yes<br>◯ No<br>◯ Prefer not to answer
Please describe any measures you have taken to increase your privacy at home. Select all that apply.	☐ I avoid having sensitive conversations around my [q1_shtobject]<br>☐ I unplug my [q1_shtobject] when it is not in use<br>☐ I have disabled some features on my [q1_shtobject] (e.g. turning off the microphone or camera)<br>☐ I have limited my privacy settings on my [q1_shtobject]<br>☐ I update my [q1_shtobject]'s software regularly<br>☐ Other (please specify)<br>☐ I have not taken any measures to protect my privacy<br>☐ Prefer not to answer
Please specify "Other":	________________________________
Do you take any measures around your [q1shtobject_other] to protect your privacy at home?	◯ Yes<br>◯ No<br>◯ Prefer not to answer
Please describe any measures you have taken to increase your privacy at home. Select all that apply.	☐ I avoid having sensitive conversations around my [q1shtobject_other]<br>☐ I unplug my [q1shtobject_other] when it is not in use<br>☐ I have disabled some features on my [q1shtobject_other] (e.g. turning off the microphone or camera)<br>☐ I have limited my privacy settings on my [q1shtobject_other]<br>☐ I update my [q1shtobject_other]'s software regularly<br>☐ Other (please specify)<br>☐ I have not taken any measures to protect my privacy<br>☐ Prefer not to answer
Please specify "Other":	________________________________

Do you believe these measures will fully protect your privacy?	◯ I believe that these measures fully protect my privacy<br>◯ These measures only protect my privacy to some extent<br>◯ I do not know if these measures will protect my privacy, but it is out of my control<br>◯ I do not think these measures protect my privacy<br>◯ Other (please specify)<br>◯ Prefer not to answer
Please specify "Other":	__________________________________
Do you believe you sacrifice your privacy for the convenience of your [q1_shtobject]?	◯ Yes<br>◯ No<br>◯ Not sure<br>◯ Prefer not to answer
Do you believe you sacrifice your privacy for the convenience of your [q1shtobject_other]?	◯ Yes<br>◯ No<br>◯ Not sure<br>◯ Prefer not to answer

Section 4: Purpose

Your [q1_shtobject] could electronically send personal information about you to the [q1_shtobject] manufacturer. These companies are able to use this information to send you advertising and product recommendations that are more in line with your interests.

The following questions explore your perspective on the collection of personal information through by your [q1_shtobject].

Your [q1shtobject_other] could electronically send personal information about you to the [q1shtobject_other] manufacturer. These companies are able to use this information to send you advertising and product recommendations that are more in line with your interests.

The following questions explore your perspective on the collection of personal information through by your [q1shtobject_other].

Do you believe your [q1_shtobject] collects personal information from you?

- ◯ My [q1_shtobject] does not collect any personal information
- ◯ My [q1_shtobject] collects some information, but only when I actively use it
- ◯ My [q1_shtobject] regularly collects my information, but I am not constantly monitored
- ◯ My [q1_shtobject] constantly collects my information/monitors me
- ◯ Other (please specify)
- ◯ Prefer not to answer

Please specify "Other":

____

Do you believe your [q1shtobject_other] collects personal information from you?

- ◯ My [q1shtobject_other] does not collect any personal information
- ◯ My [q1shtobject_other] collects some information, but only when I actively use it
- ◯ My [q1shtobject_other] regularly collects my information, but I am not constantly monitored
- ◯ My [q1shtobject_other] constantly collects my information/monitors me
- ◯ Other (please specify)
- ◯ Prefer not to answer

Please specify "Other":

____

What kind of information do you think is collected? Select all that apply.

- ☐ Search history
- ☐ Location
- ☐ Voice recordings
- ☐ Video recordings
- ☐ Timing of use of my [q1_shtobject]
- ☐ Motion (your movement within your home)
- ☐ Not sure
- ☐ Other (please specify):
- ☐ Prefer not to answer

Please specify "Other":

____

What kind of information do you think is collected? Select all that apply.	☐ Search history<br>☐ Location<br>☐ Voice recordings<br>☐ Video recordings<br>☐ Timing of use of my [q1shtobject_other]<br>☐ Motion (your movement within your home)<br>☐ Not sure<br>☐ Other (please specify):<br>☐ Prefer not to answer
Please specify "Other":	__________________________________
Why do you believe your personal information is collected? Select all that apply.	☐ My [q1_shtobject] collects my information, but it is essential for its function<br>☐ My [q1_shtobject] likely collects personal information that is unnecessary for its function, but I do not know why<br>☐ My [q1_shtobject] collects my information both for its function and for the [q1_shtobject] producers (i.e. companies) to store and use for marketing purposes<br>☐ My [q1_shtobject] collects my information so the government can monitor me<br>☐ My [q1_shtobject] collects my information so my family can monitor me<br>☐ My [q1_shtobject] collects my information for healthcare reasons<br>☐ Not sure<br>☐ Other (please specify)<br>☐ Prefer not to answer
Please specify "Other":	__________________________________
Why do you believe your personal information is collected? Select all that apply.	☐ My [q1shtobject_other] collects my information, but it is essential for its function<br>☐ My [q1shtobject_other] likely collects personal information that is unnecessary for its function, but I do not know why<br>☐ My [q1shtobject_other] collects my information both for its function and for the [q1shtobject_other] producers (i.e. companies) to store and use for marketing purposes<br>☐ My [q1shtobject_other] collects my information so the government can monitor me<br>☐ My [q1shtobject_other] collects my information so my family can monitor me<br>☐ My [q1shtobject_other] collects my information for healthcare reasons<br>☐ Not sure<br>☐ Other (please specify)<br>☐ Prefer not to answer
Please specify "Other":	__________________________________
Have you read the Terms and Conditions for your [q1_shtobject] in detail?	◯ Yes<br>◯ No<br>◯ Not sure<br>◯ Prefer not to answer

Have you read the Terms and Conditions for your [q1shtobject_other] in detail?

- ◯ Yes
- ◯ No
- ◯ Not sure
- ◯ Prefer not to answer

Section 5: Risk

There are always risks associated with using technology, ranging from inconveniences to health hazards. The following questions explore your views on the risks associated with smart home technology.

When you received your [q1_shtobject], were you concerned about any risks? These risks could include privacy loss, malfunction, or just hassle.	◯ Yes<br>◯ No<br>◯ Prefer not to answer
When you received your [q1shtobject_other], were you concerned about any risks? These risks could include privacy loss, malfunction, or just hassle.	◯ Yes<br>◯ No<br>◯ Prefer not to answer
Which potential risks were you concerned about? Select all that apply.	☐ I was concerned with losing my personal information<br>☐ I was concerned I could be monitored<br>☐ I was concerned my [q1_shtobject] would underperform<br>☐ I was concerned my [q1_shtobject] would malfunction<br>☐ I was concerned that my [q1_shtobject] could be hacked<br>☐ Other (please specify)<br>☐ Prefer not to answer
Please specify "Other":	__________________________________
Which potential risks were you concerned about? Select all that apply.	☐ I was concerned with losing my personal information<br>☐ I was concerned I could be monitored<br>☐ I was concerned my [q1shtobject_other] would underperform<br>☐ I was concerned my [q1shtobject_other] would malfunction<br>☐ I was concerned that my [q1shtobject_other] could be hacked<br>☐ Other (please specify)<br>☐ Prefer not to answer
Please specify "Other":	__________________________________
Do you think that the risks could be reduced by new policies, new laws, or by the device designers?	◯ Yes<br>◯ No<br>◯ Not sure<br>◯ Other (please specify)<br>◯ Prefer not to answer
Please specify "Other":	__________________________________
Have your perceptions of these risks changed from when you received your [q1_shtobject] until now?	◯ I am more concerned about the risks associated with my [q1_shtobject]<br>◯ I am less concerned about the risks associated with my [q1_shtobject]<br>◯ My concerns have not changed<br>◯ Other (please specify)<br>◯ Prefer not to answer
Please specify "Other":	__________________________________

Have your perceptions of these risks changed from when you received your [q1shtobject_other] until now?	◯ I am more concerned about the risks associated with my [q1shtobject_other]<br>◯ I am less concerned about the risks associated with my [q1shtobject_other]<br>◯ My concerns have not changed<br>◯ Other (please specify)<br>◯ Prefer not to answer
Please specify "Other":	__________________________________
What are the benefits of your [q1_shtobject], if any? Select all that apply.	☐ Low cost<br>☐ Enhanced daily convenience<br>☐ Health benefits<br>☐ Increased independence<br>☐ Enhanced safety<br>☐ Enhanced security<br>☐ No benefits<br>☐ Other (please specify)<br>☐ Prefer not to answer
Please specify "Other":	__________________________________
What are the benefits of your [q1shtobject_other], if any? Select all that apply.	☐ Low cost<br>☐ Enhanced daily convenience<br>☐ Health benefits<br>☐ Increased independence<br>☐ Enhanced safety<br>☐ Enhanced security<br>☐ No benefits<br>☐ Other (please specify)<br>☐ Prefer not to answer
Please specify "Other":	__________________________________
Did the benefits of your [q1_shtobject] outweigh the concerns your selected above?	◯ Yes<br>◯ No<br>◯ Not sure<br>◯ Prefer not to answer
Did the benefits of your [q1shtobject_other] outweigh the concerns your selected above?	◯ Yes<br>◯ No<br>◯ Not sure<br>◯ Prefer not to answer

Section 6: Safety

Smart home objects can impact your safety at home in a variety of ways. The following questions explore how you feel about safety and your [q1_shtobject].

Smart home objects can impact your safety at home in a variety of ways. The following questions explore how you feel about safety and your [q1shtobject_other].

Does your [q1_shtobject] make you feel safer in your home?	◯ Yes<br>◯ No<br>◯ Sometimes<br>◯ Not sure<br>◯ Prefer not to answer
Does your [q1shtobject_other] make you feel safer in your home?	◯ Yes<br>◯ No<br>◯ Sometimes<br>◯ Not sure<br>◯ Prefer not to answer
Why you feel safer? Select all that apply.	☐ My [q1_shtobject] could detect and connect me to emergency services quickly if I am in danger (e.g., fall, heart attack, violence)<br>☐ I could connect with family (or vice versa) more easily during an emergency<br>☐ Other (please specify)<br>☐ Prefer not to answer
Please specify "Other":	______________________________
Why you feel safer? Select all that apply.	☐ My [q1shtobject_other] could detect and connect me to emergency services quickly if I am in danger (e.g., fall, heart attack, violence)<br>☐ I could connect with family (or vice versa) more easily during an emergency<br>☐ Other (please specify)<br>☐ Prefer not to answer
Please specify "Other":	______________________________
