# Supplementary material for: Examining Canadian Smart Home Technology Users’ Perceptions of Privacy, Risks, Safety, and Data Collection Purposes: Cross-Sectional National Survey
Source: JMIR Form Res. 2026 Sep 23;10:e70638. doi: 10.2196/70638 (PMC13600659; doi:10.2196/70638)
Supplement: Supplementary file 2 [file formative-v10-e70638-s002.pdf]

Preliminary notes:

- "Devices" and "Objects" are used in the literature to describe Smart technologies like smart fridges, watches, etc. Therefore, their use has been employed within this survey in order to differentiate from non-smart objects/devices such as televisions or cell phones.

- Final number of records after removal of duplicates used for data analysis: 886
- Responses indicating they own smart home objects: 881
- Responses indicating they don't own smart home objects: 5
    - Age: 30-39 [1], 40-49 [1], 70-79 [3]
    - Education: Graduate or professional degree [3], Secondary school (High school) [1], College diploma [1]
    - Ethnicity: North American White [4], European heritage [1]
    - Gender: Male [1], Female [4]
    - Income: Over $150,000 [1], $75,000 to $100,000 [1], $25,000 to $50,000 [3],

Further data analysis is done on 881 records that indicated that they own smart home objects.

Groups:

- Smart speakers: Google home, Amazon Alexa smart speaker, Google Nest/Google Nest Hub
- Security systems: Smart lock system, Amazon ring, Motion sensor
- Safety sensors: Fall detection sensor, Emergency contact sensor
- Other appliances: Smart lights, Smart fridge, Smart faucet,Temperature sensor, Humidity sensor, Other

Demographic Information

Q: What is your age, in years?

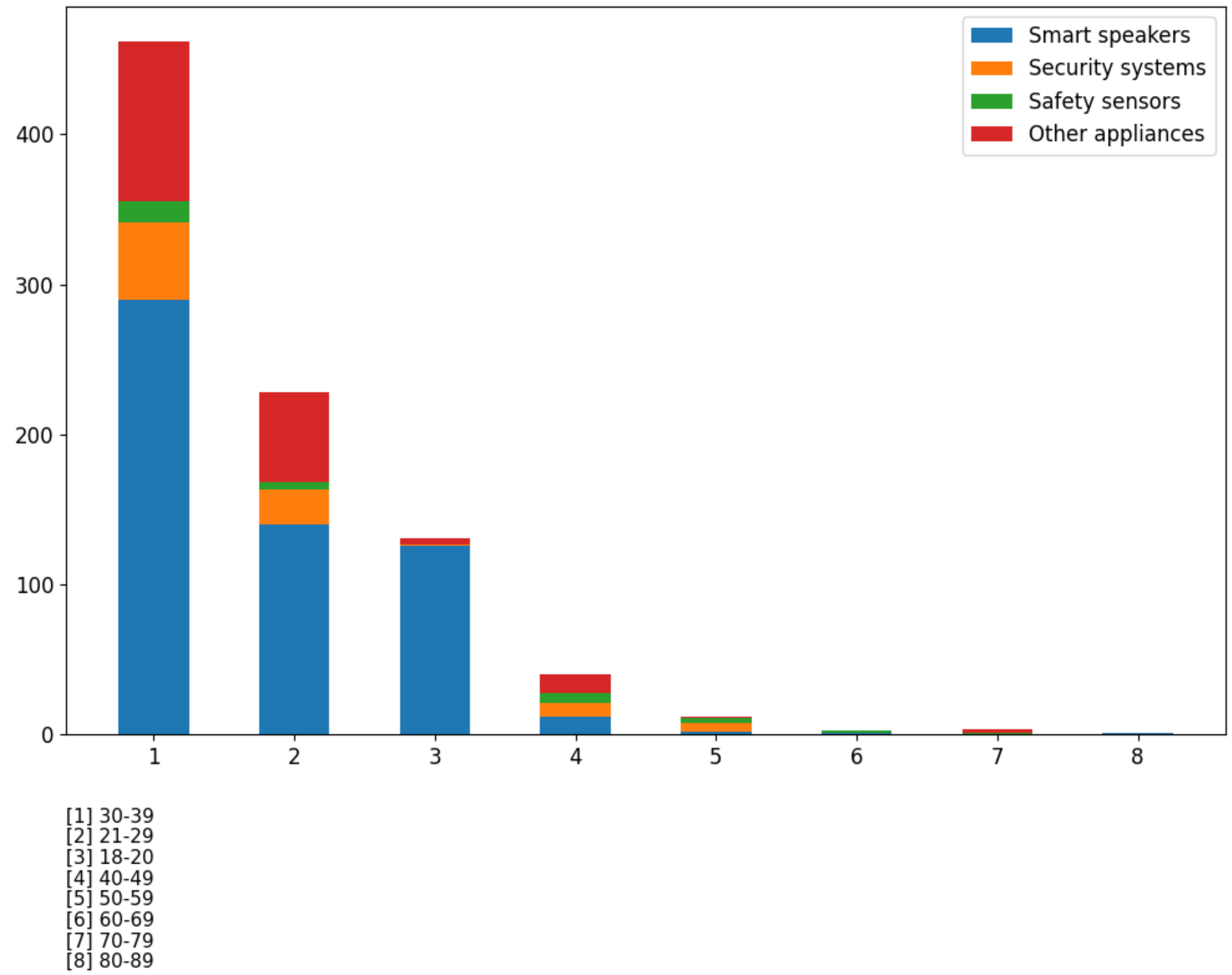

Figure S1

- 30-39 : {'Smart speakers': 290, 'Security systems': 51, 'Safety sensors': 14, 'Other appliances': 107}
- 21-29 : {'Smart speakers': 140, 'Security systems': 23, 'Safety sensors': 5, 'Other appliances': 60}
- 18-20 : {'Smart speakers': 126, 'Security systems': 1, 'Other appliances': 4}
- 40-49 : {'Smart speakers': 12, 'Security systems': 9, 'Safety sensors': 7, 'Other appliances': 12}
- 50-59 : {'Smart speakers': 2, 'Security systems': 6, 'Safety sensors': 3, 'Other appliances': 1}
- 60-69 : {'Smart speakers': 1, 'Safety sensors': 2}

- 70-79 : {'Safety sensors': 1, 'Other appliances': 3}
- 80-89 : {'Smart speakers': 1}

Q: What is your highest level of completed education?

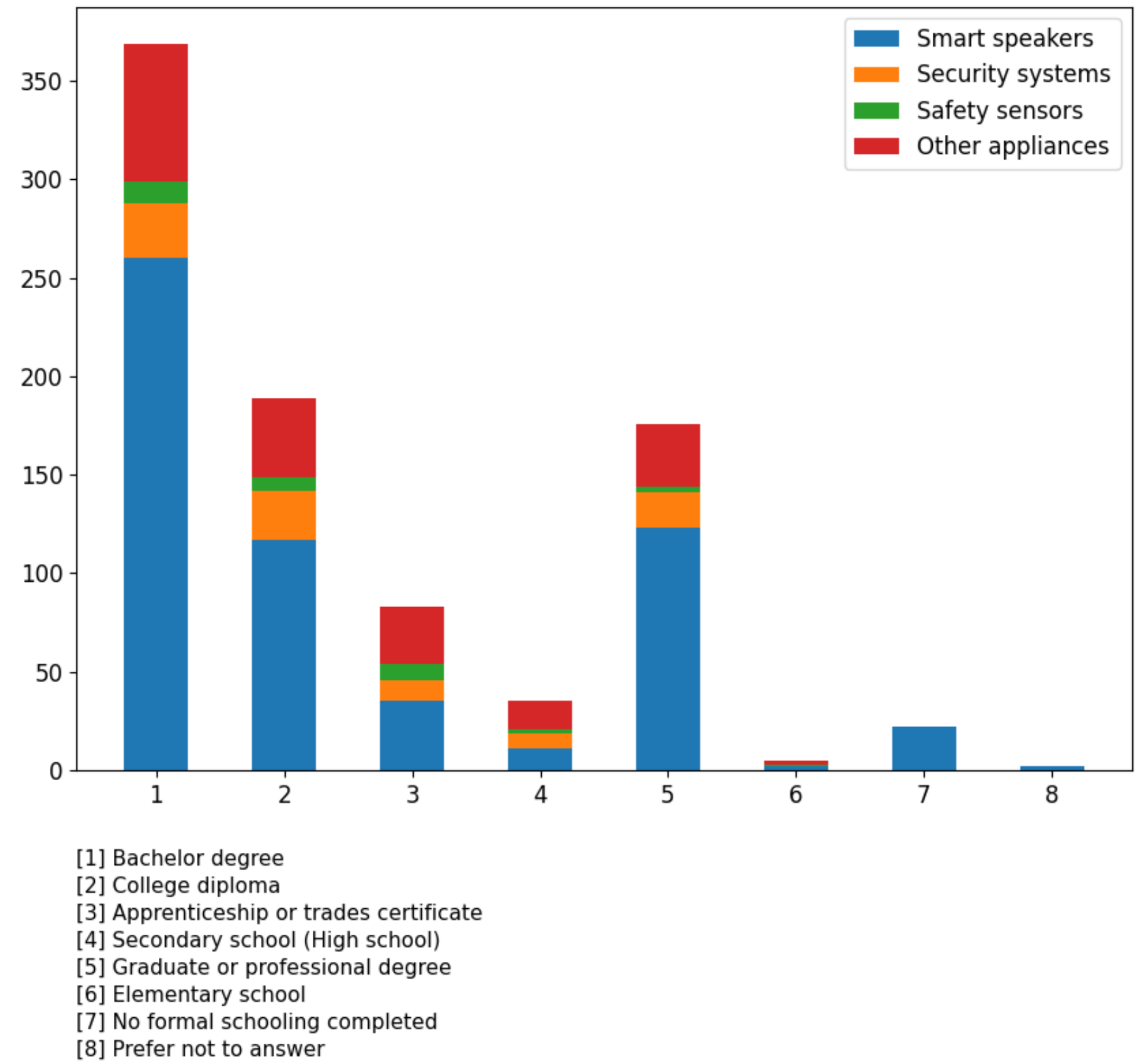

Figure S2

- Bachelor degree : {'Smart speakers': 260, 'Security systems': 28, 'Safety sensors': 11, 'Other appliances': 70}
- College diploma : {'Smart speakers': 117, 'Security systems': 25, 'Safety sensors': 7, 'Other appliances': 40}
- Apprenticeship or trades certificate : {'Smart speakers': 35, 'Security systems': 11, 'Safety sensors': 8, 'Other appliances': 29}
- Secondary school (High school) : {'Smart speakers': 11, 'Security systems': 8, 'Safety sensors': 2, 'Other appliances': 14}
- Graduate or professional degree : {'Smart speakers': 123, 'Security systems': 18, 'Safety sensors': 3, 'Other appliances': 32}

- Elementary school : {'Smart speakers': 2, 'Safety sensors': 1, 'Other appliances': 2}
- No formal schooling completed : {'Smart speakers': 22}
- Prefer not to answer : {'Smart speakers': 2}

Q: What is your annual income, in Canadian dollars (CAD)?

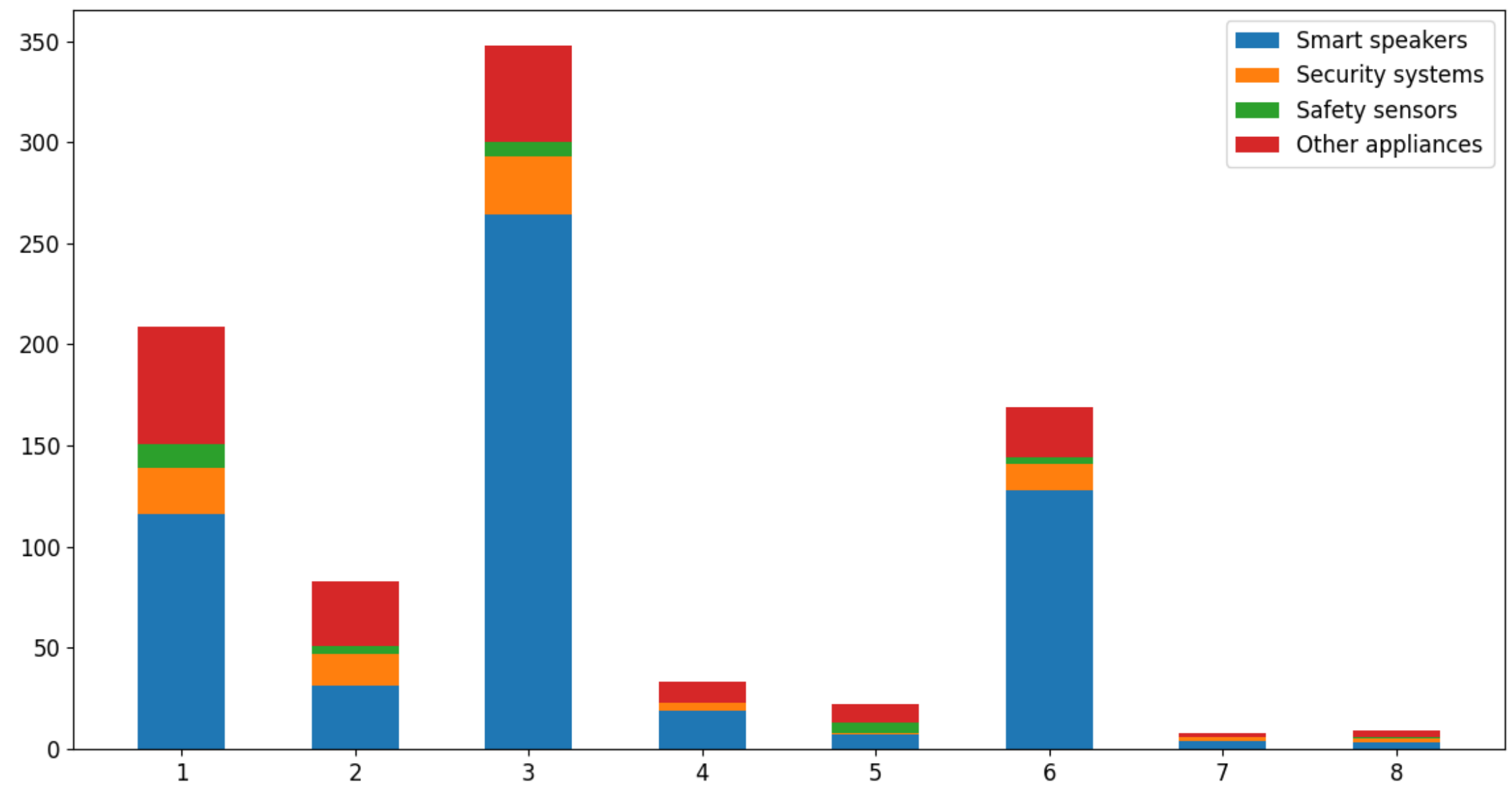

[1] $75,000 to $100,000
[2] $100,000 to $125,000
[3] $50,000 to $75,000
[4] $125,000 to $150,000
[5] Less than $25,000
[6] $25,000 to $50,000
[7] Prefer not to answer
[8] Over $150,000

Figure S3

- $75,000 to $100,000 : {'Smart speakers': 116, 'Security systems': 23, 'Safety sensors': 12, 'Other appliances': 58}
- $100,000 to $125,000 : {'Smart speakers': 31, 'Security systems': 16, 'Safety sensors': 4, 'Other appliances': 32}
- $50,000 to $75,000 : {'Smart speakers': 264, 'Security systems': 29, 'Safety sensors': 7, 'Other appliances': 48}
- $125,000 to $150,000 : {'Smart speakers': 19, 'Security systems': 4, 'Other appliances': 10}
- Less than $25,000 : {'Smart speakers': 7, 'Security systems': 1, 'Safety sensors': 5, 'Other appliances': 9}
- $25,000 to $50,000 : {'Smart speakers': 128, 'Security systems': 13, 'Safety sensors': 3, 'Other appliances': 25}
- Prefer not to answer : {'Smart speakers': 4, 'Security systems': 2, 'Other appliances': 2}
- Over $150,000 : {'Smart speakers': 3, 'Security systems': 2, 'Safety sensors': 1, 'Other appliances': 3}

Q: What is your ethnicity?

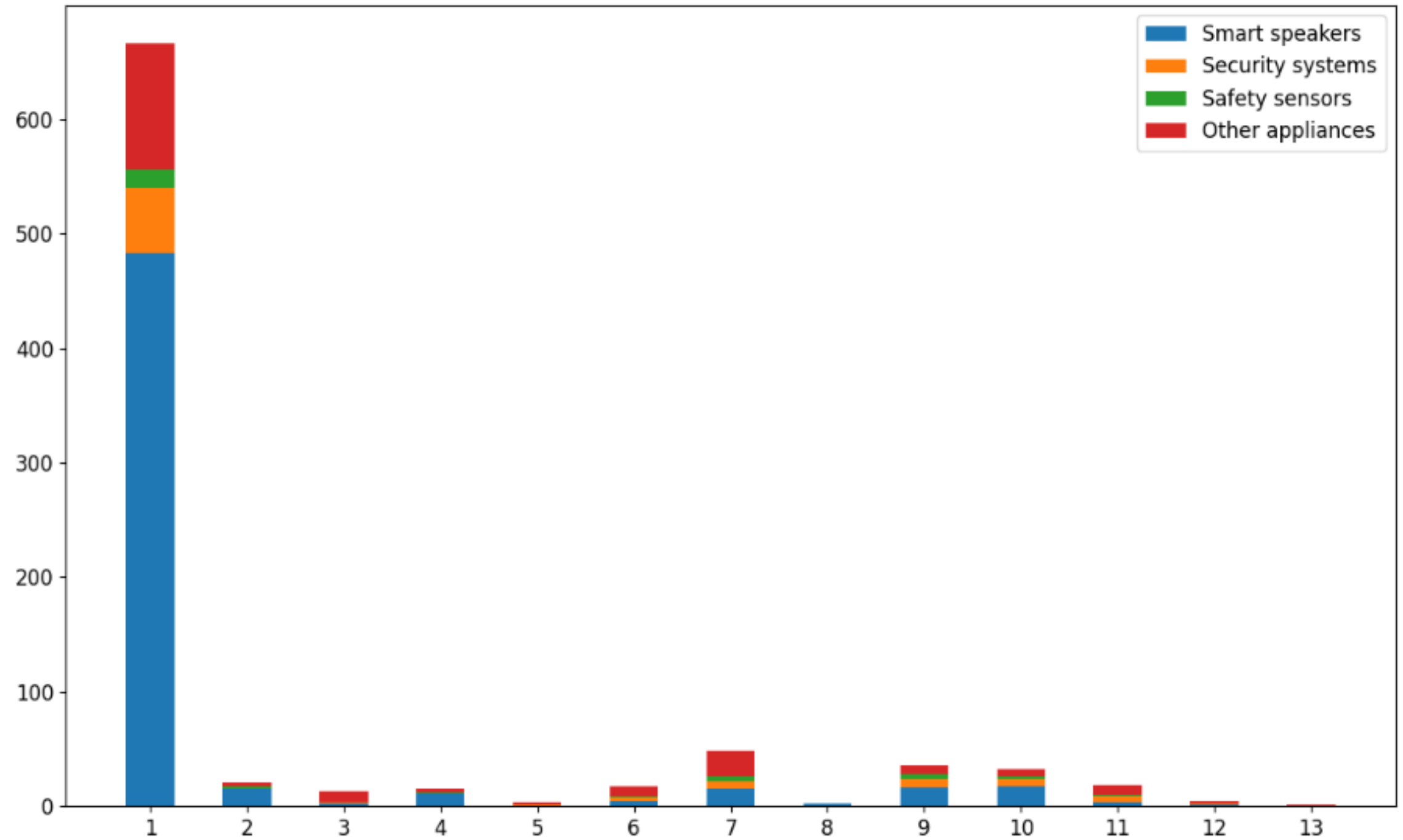

[1] North American White
[2] African heritage
[3] East Asian heritage
[4] South Asian heritage
[5] Prefer not to answer
[6] Central American Indigenous heritage
[7] North American Indigenous heritage
[8] South East Asian heritage
[9] South American Indigenous heritage
[10] European heritage
[11] Latin American
[12] Middle Eastern heritage
[13] Central Asian heritage

Figure S4

- North American White : {'Smart speakers': 483, 'Security systems': 57, 'Safety sensors': 16, 'Other appliances': 110}
- African heritage : {'Smart speakers': 15, 'Safety sensors': 2, 'Other appliances': 4}
- East Asian heritage : {'Smart speakers': 2, 'Security systems': 2, 'Other appliances': 9}
- South Asian heritage : {'Smart speakers': 11, 'Safety sensors': 1, 'Other appliances': 3}
- Prefer not to answer : {'Security systems': 1, 'Other appliances': 3}
- Central American Indigenous heritage : {'Smart speakers': 5, 'Security systems': 3, 'Safety sensors': 1, 'Other appliances': 8}
- North American Indigenous heritage : {'Smart speakers': 15, 'Security systems': 7, 'Safety sensors': 4, 'Other appliances': 23}
- South East Asian heritage : {'Smart speakers': 2}
- South American Indigenous heritage : {'Smart speakers': 16, 'Security systems': 8, 'Safety sensors': 4, 'Other appliances': 8}
- European heritage : {'Smart speakers': 18, 'Security systems': 6, 'Safety sensors': 2, 'Other appliances': 7}
- Latin American : {'Smart speakers': 4, 'Security systems': 5, 'Safety sensors': 1, 'Other appliances': 9}
- Middle Eastern heritage : {'Smart speakers': 1, 'Security systems': 1, 'Safety sensors': 1, 'Other appliances': 2}
- Central Asian heritage : {'Other appliances': 1}

Q: What is your gender identity?

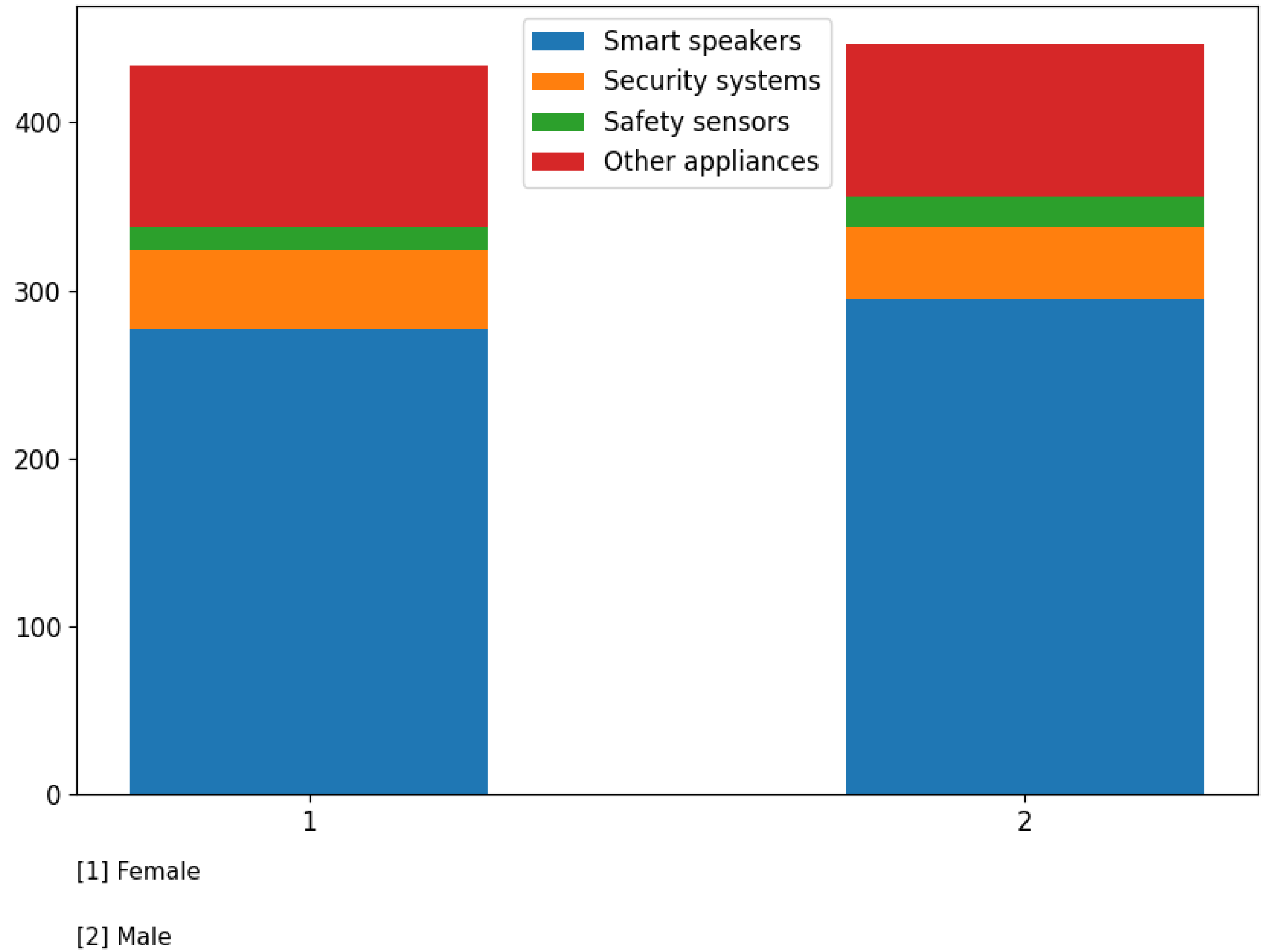

Figure S5

- Female : {'Smart speakers': 277, 'Security systems': 47, 'Safety sensors': 14, 'Other appliances': 96}
- Male : {'Smart speakers': 295, 'Security systems': 43, 'Safety sensors': 18, 'Other appliances': 91}

Smart Home Object Information

Q: Why did you purchase your smart object?

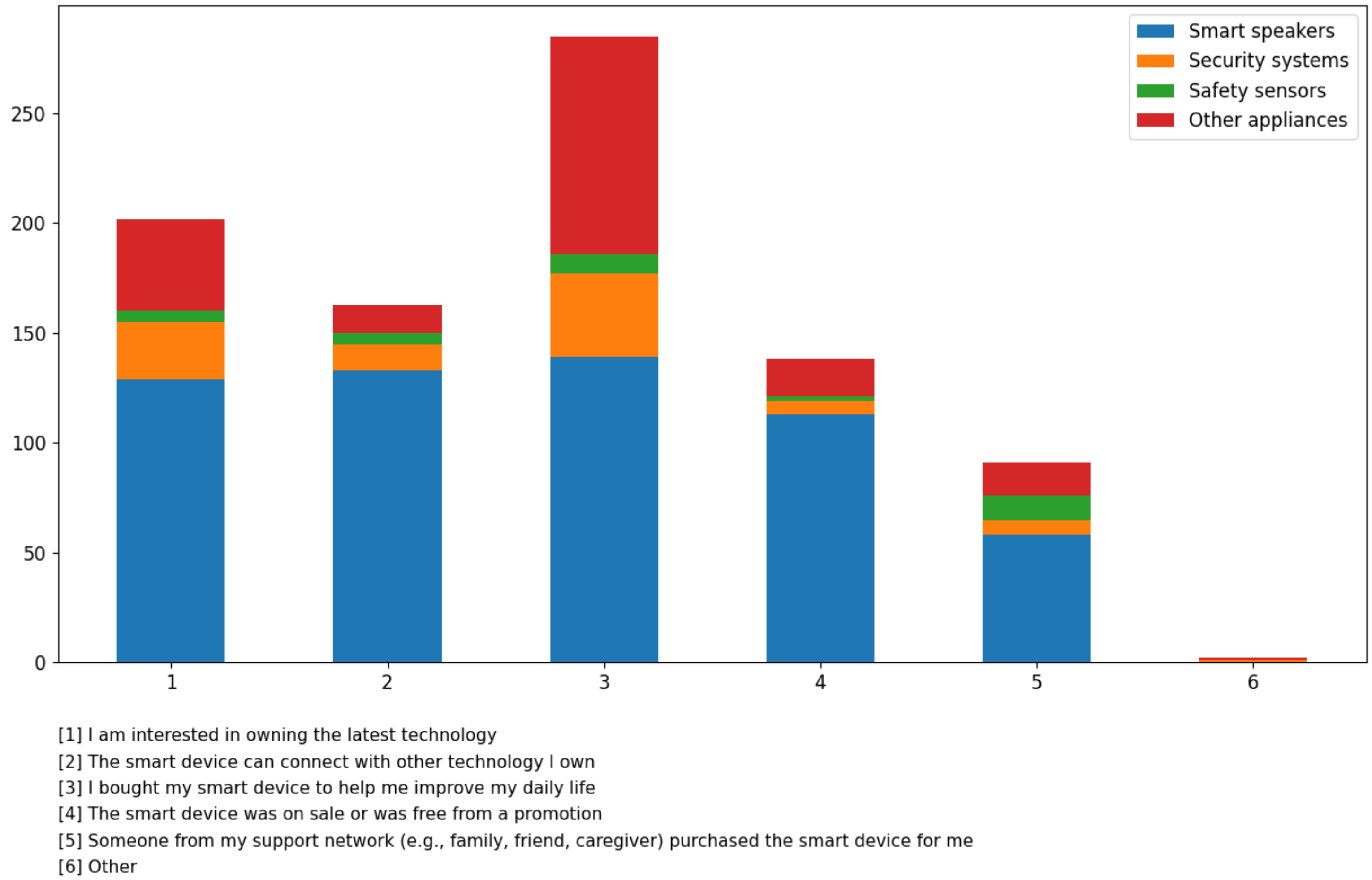

Figure S6

- I am interested in owning the latest technology : {'Smart speakers': 129, 'Security systems': 26, 'Safety sensors': 5, 'Other appliances': 42}

- The smart device can connect with other technology I own : {'Smart speakers': 133, 'Security systems': 12, 'Safety sensors': 5, 'Other appliances': 13}
- I bought my smart device to help me improve my daily life : {'Smart speakers': 139, 'Security systems': 38, 'Safety sensors': 9, 'Other appliances': 99}
- The smart device was on sale or was free from a promotion : {'Smart speakers': 113, 'Security systems': 6, 'Safety sensors': 2, 'Other appliances': 17}
- Someone from my support network (e.g., family, friend, caregiver) purchased the smart device for me : {'Smart speakers': 58, 'Security systems': 7, 'Safety sensors': 11, 'Other appliances': 15}
- Other : {'Security systems': 1, 'Other appliances': 1}

Other:

- Recommended by police because of a neighbourhood disruption
- part of new heat pump

Q: How long have you owned your smart object?

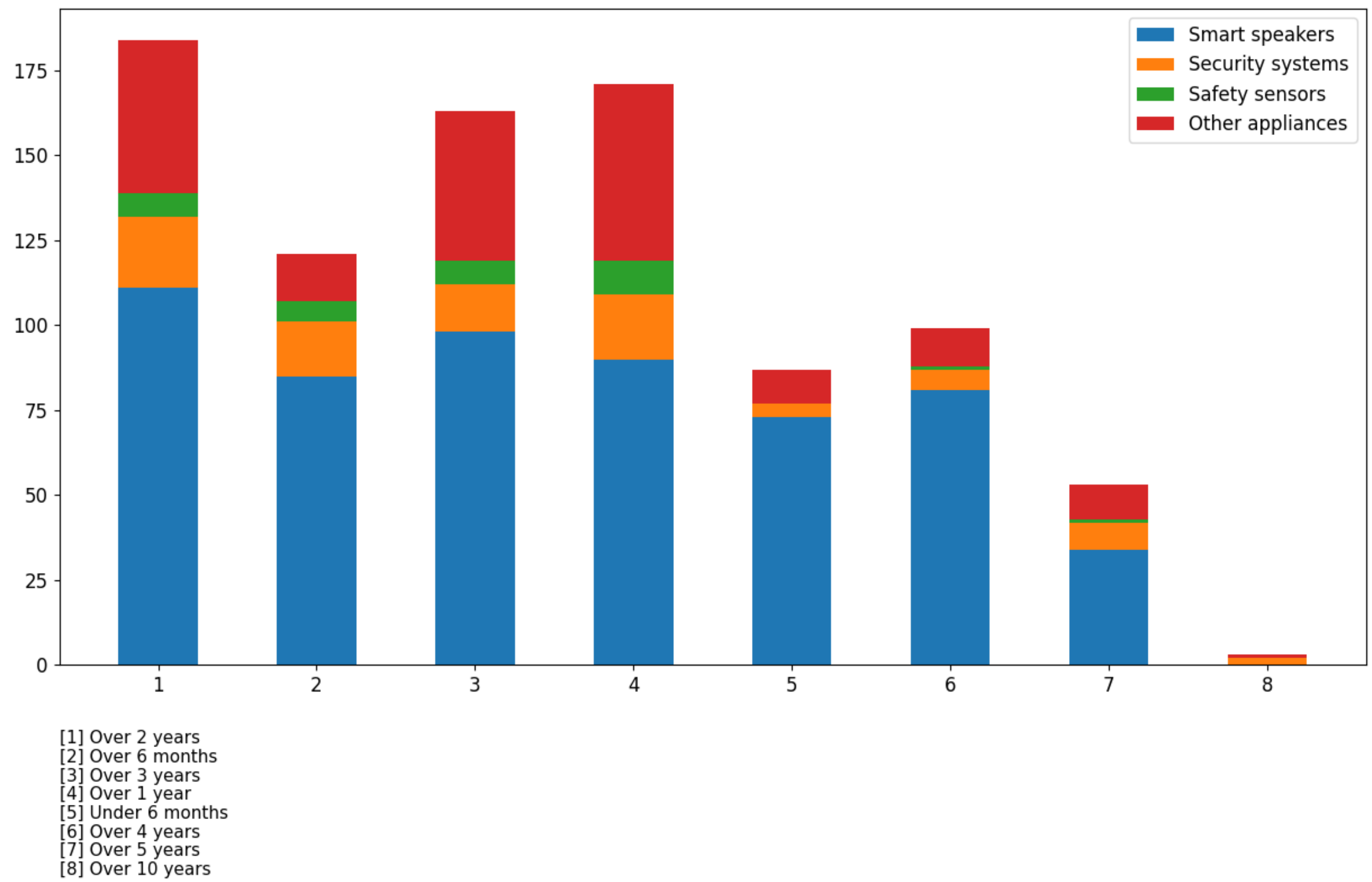

Figure S7

- Over 2 years : {'Smart speakers': 111, 'Security systems': 21, 'Safety sensors': 7, 'Other appliances': 45}
- Over 6 months : {'Smart speakers': 85, 'Security systems': 16, 'Safety sensors': 6, 'Other appliances': 14}
- Over 3 years : {'Smart speakers': 98, 'Security systems': 14, 'Safety sensors': 7, 'Other appliances': 44}
- Over 1 year : {'Smart speakers': 90, 'Security systems': 19, 'Safety sensors': 10, 'Other appliances': 52}
- Under 6 months : {'Smart speakers': 73, 'Security systems': 4, 'Other appliances': 10}
- Over 4 years : {'Smart speakers': 81, 'Security systems': 6, 'Safety sensors': 1, 'Other appliances': 11}

- Over 5 years : {'Smart speakers': 34, 'Security systems': 8, 'Safety sensors': 1, 'Other appliances': 10}
- Over 10 years : {'Security systems': 2, 'Other appliances': 1}

Q: Do you use your smart object more than anyone else who spends time in your home?

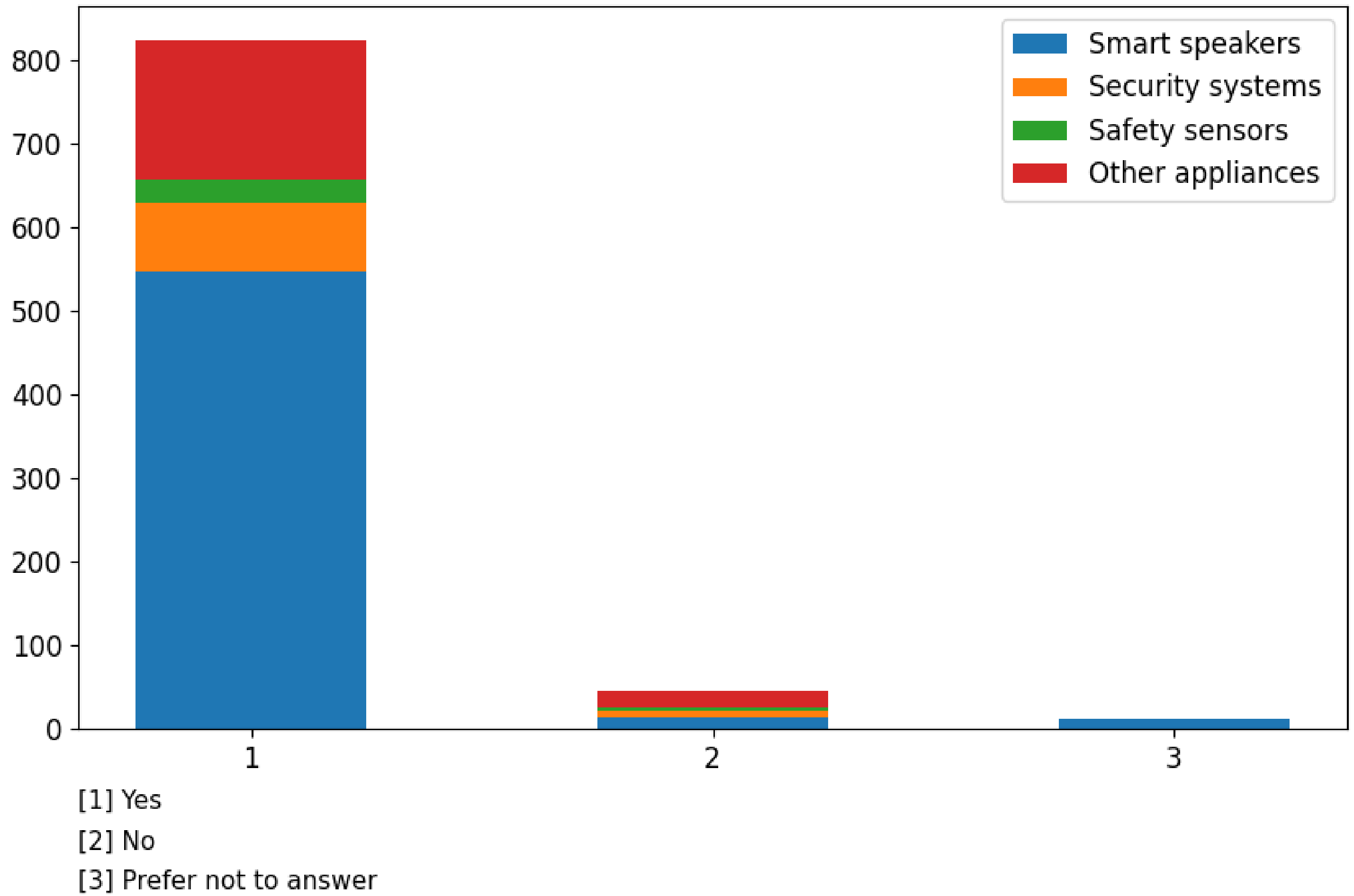

Figure S8

- Yes : {'Smart speakers': 546, 'Security systems': 83, 'Safety sensors': 28, 'Other appliances': 166}
- No : {'Smart speakers': 14, 'Security systems': 7, 'Safety sensors': 4, 'Other appliances': 21}
- Prefer not to answer : {'Smart speakers': 12}

Q: If not you, who uses it the most?

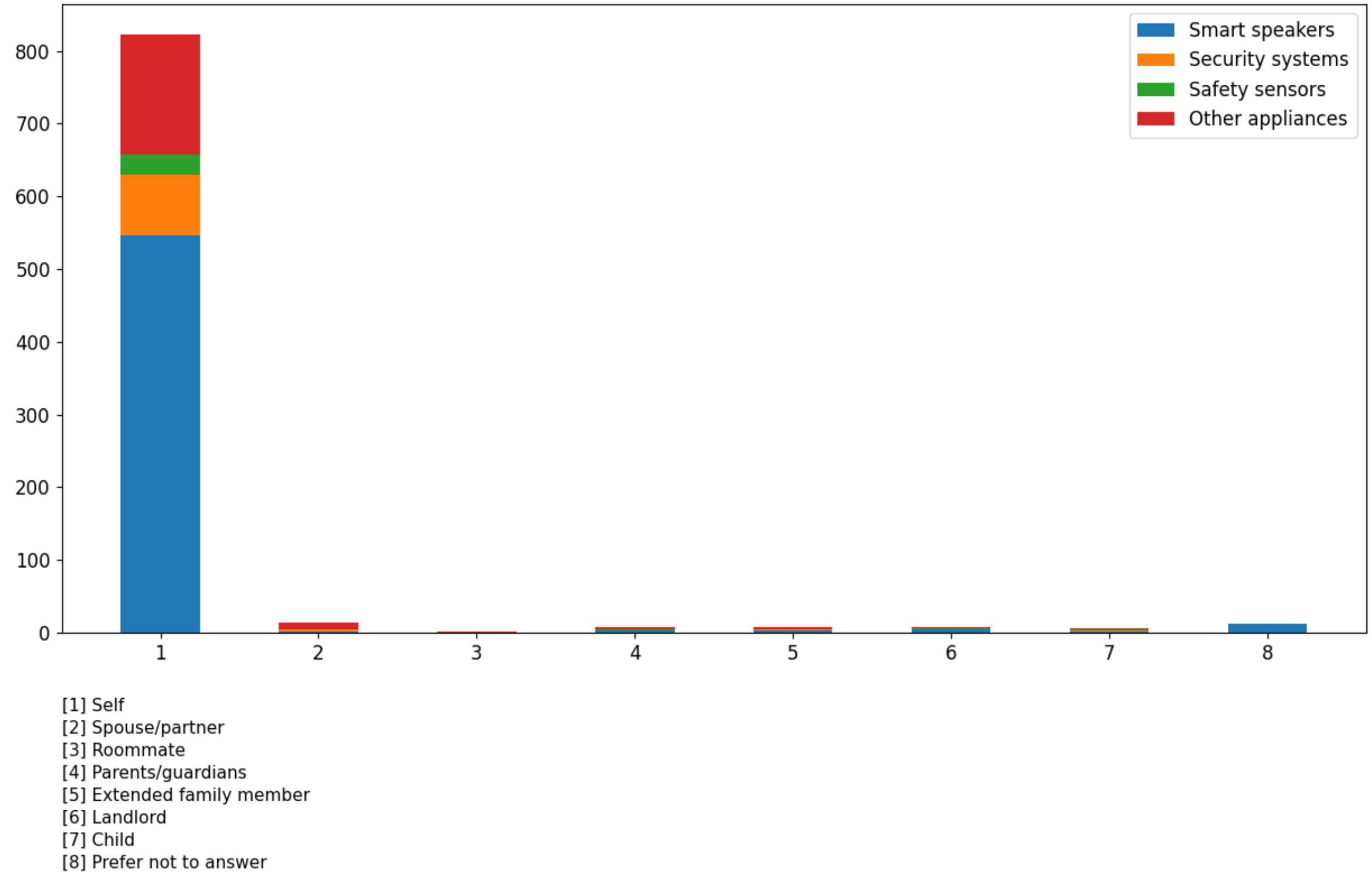

Figure S9

- Self : {'Smart speakers': 546, 'Security systems': 83, 'Safety sensors': 28, 'Other appliances': 166}
- Spouse/partner : {'Smart speakers': 1, 'Security systems': 4, 'Other appliances': 9}
- Roommate : {'Other appliances': 2}
- Parents/guardians : {'Smart speakers': 3, 'Safety sensors': 2, 'Other appliances': 3}
- Extended family member : {'Smart speakers': 3, 'Security systems': 2, 'Other appliances': 3}
- Landlord : {'Smart speakers': 5, 'Safety sensors': 1, 'Other appliances': 1}
- Child : {'Smart speakers': 2, 'Security systems': 1, 'Safety sensors': 1, 'Other appliances': 2}

- Prefer not to answer : {'Smart speakers': 12, 'Other appliances': 1}

Section 3: Privacy

Q: Select the option that best matches your feelings about privacy in regard to your smart object.

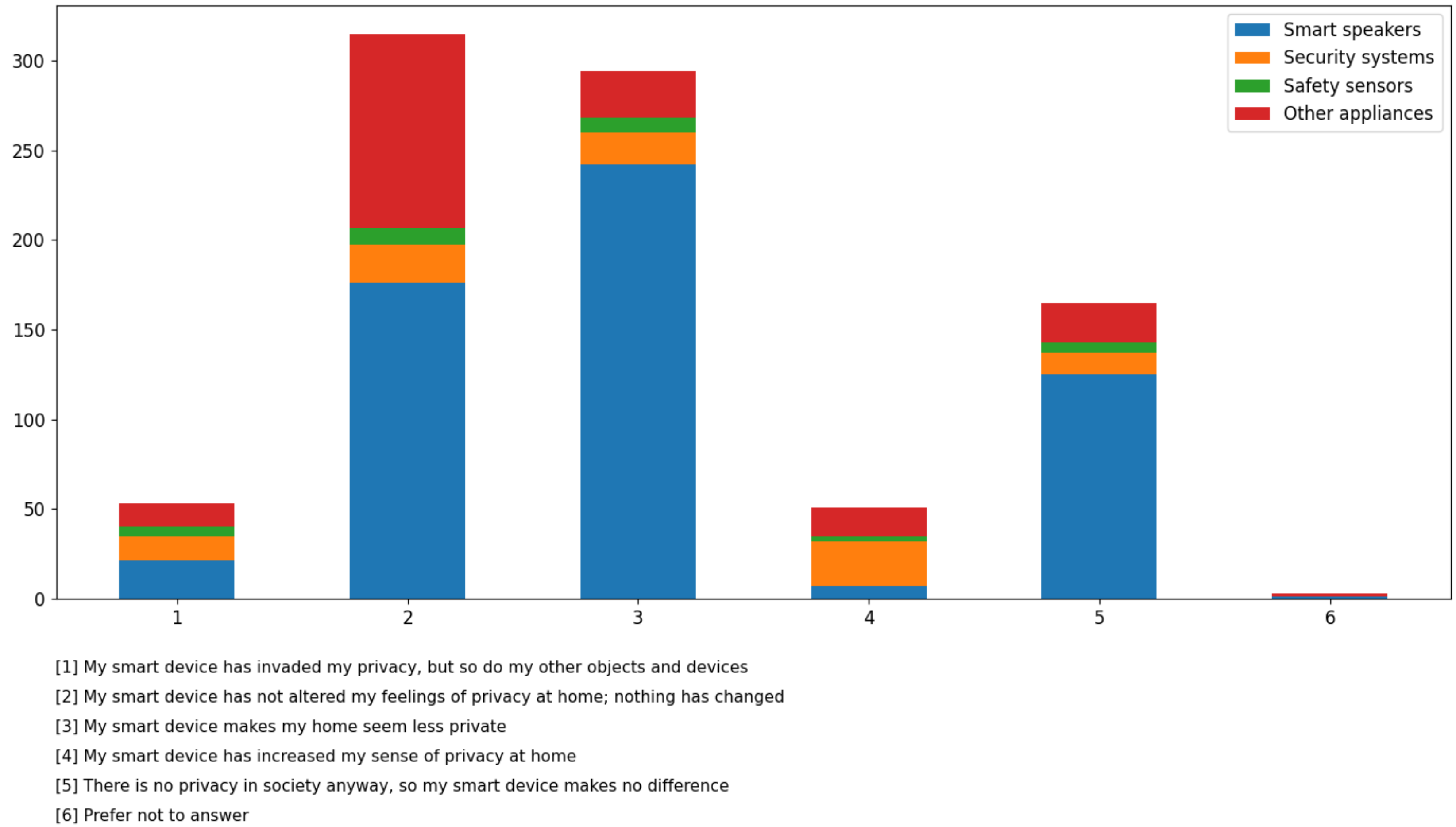

Figure S10

- My smart device has invaded my privacy, but so do my other objects and devices : {'Smart speakers': 21, 'Security systems': 14, 'Safety sensors': 5, 'Other appliances': 13}

- My smart device has not altered my feelings of privacy at home; nothing has changed : {'Smart speakers': 176, 'Security systems': 21, 'Safety sensors': 10, 'Other appliances': 108}
- My smart device makes my home seem less private : {'Smart speakers': 242, 'Security systems': 18, 'Safety sensors': 8, 'Other appliances': 26}
- My smart device has increased my sense of privacy at home : {'Smart speakers': 7, 'Security systems': 25, 'Safety sensors': 3, 'Other appliances': 16}
- There is no privacy in society anyway, so my smart device makes no difference : {'Smart speakers': 125, 'Security systems': 12, 'Safety sensors': 6, 'Other appliances': 22}
- Prefer not to answer : {'Smart speakers': 1, 'Other appliances': 2}

Q: Which of the following statements describes your concerns over privacy and your smart object? Select all that apply.

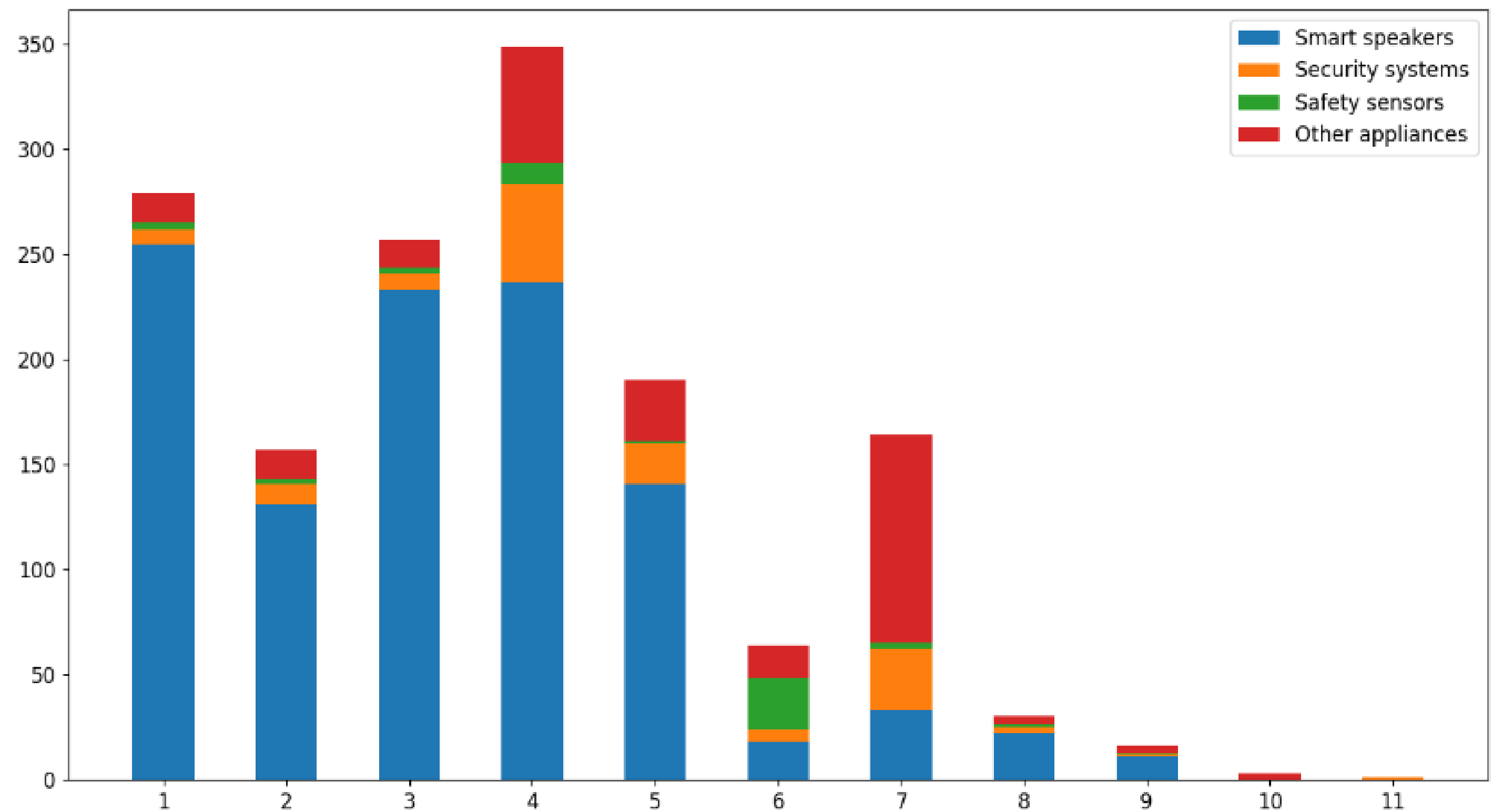

[1] Companies can monitor me through my smart device
[2] Law enforcement can monitor me
[3] The government can monitor me
[4] My private information could be collected and shared without my authorization
[5] My smart device could be hacked
[6] Family members could spy on me with my smart device
[7] No concerns
[8] My landlord could spy on me with my smart device
[9] Roommates could spy on me with my smart device
[10] Prefer not to answer
[11] Other

Figure S11

- Companies can monitor me through my smart device : {'Smart speakers': 255, 'Security systems': 7, 'Safety sensors': 3, 'Other appliances': 14}
- Law enforcement can monitor me : {'Smart speakers': 131, 'Security systems': 10, 'Safety sensors': 2, 'Other appliances': 14}
- The government can monitor me : {'Smart speakers': 233, 'Security systems': 8, 'Safety sensors': 3, 'Other appliances': 13}
- My private information could be collected and shared without my authorization : {'Smart speakers': 237, 'Security systems': 46, 'Safety sensors': 11, 'Other appliances': 55}
- My smart device could be hacked : {'Smart speakers': 141, 'Security systems': 19, 'Safety sensors': 1, 'Other appliances': 29}
- Family members could spy on me with my smart device : {'Smart speakers': 18, 'Security systems': 6, 'Safety sensors': 24, 'Other appliances': 16}
- No concerns : {'Smart speakers': 33, 'Security systems': 29, 'Safety sensors': 3, 'Other appliances': 99}
- My landlord could spy on me with my smart device : {'Smart speakers': 22, 'Security systems': 3, 'Safety sensors': 1, 'Other appliances': 4}
- Roommates could spy on me with my smart device : {'Smart speakers': 11, 'Security systems': 1, 'Safety sensors': 1, 'Other appliances': 3}
- Prefer not to answer : {'Other appliances': 3}
- Other : {'Security systems': 1}

Other:

- Family members can see me walking in with suprirse birthday presents!

Q: Do you take any measures around your smart object to protect your privacy at home?

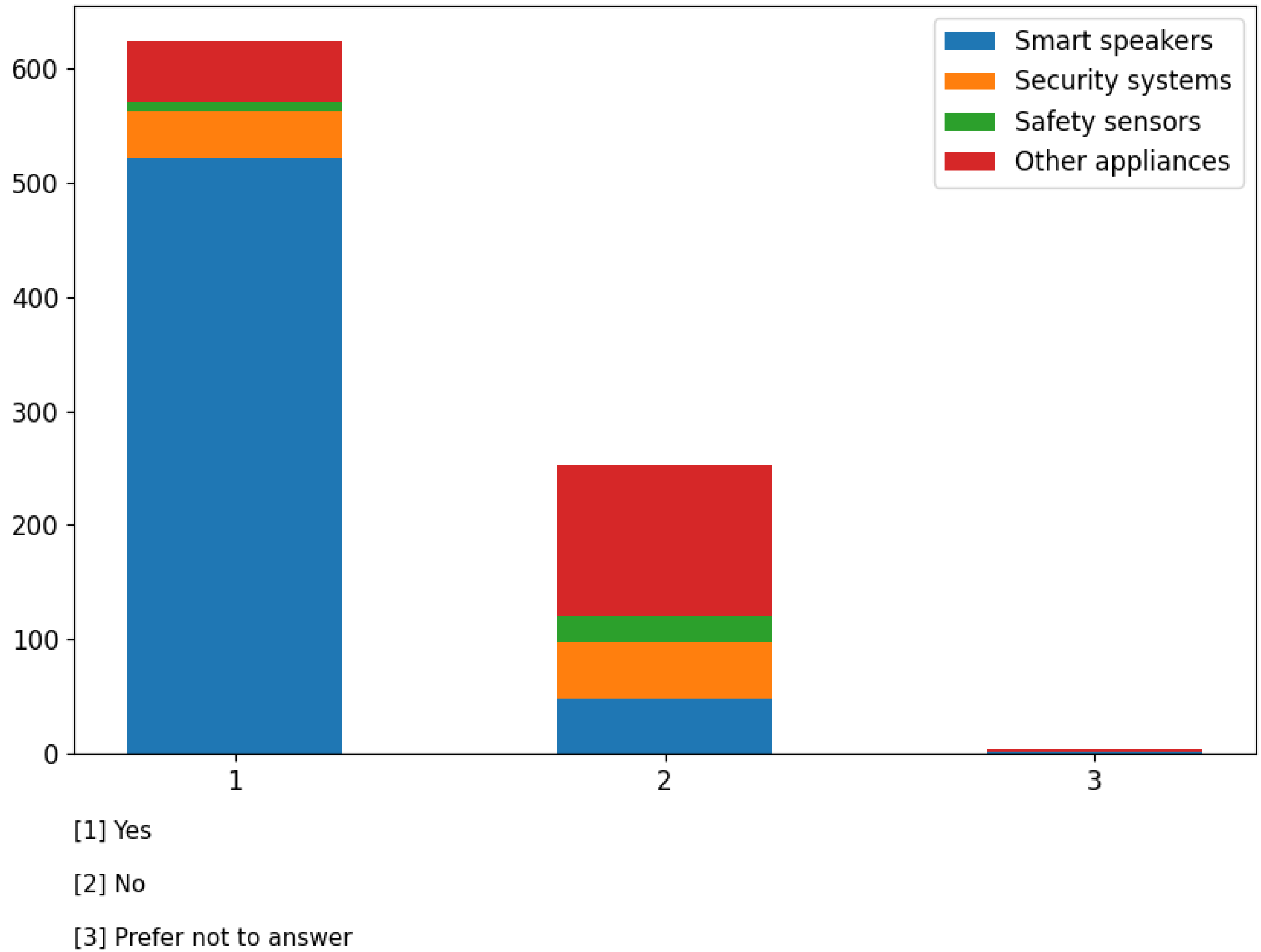

Figure S12

- Yes : {'Smart speakers': 522, 'Security systems': 41, 'Safety sensors': 8, 'Other appliances': 53}

- No : {'Smart speakers': 48, 'Security systems': 49, 'Safety sensors': 24, 'Other appliances': 132}
- Prefer not to answer : {'Smart speakers': 2, 'Other appliances': 2}

Q: Please describe any measures you have taken to increase your privacy at home. Select all that apply.

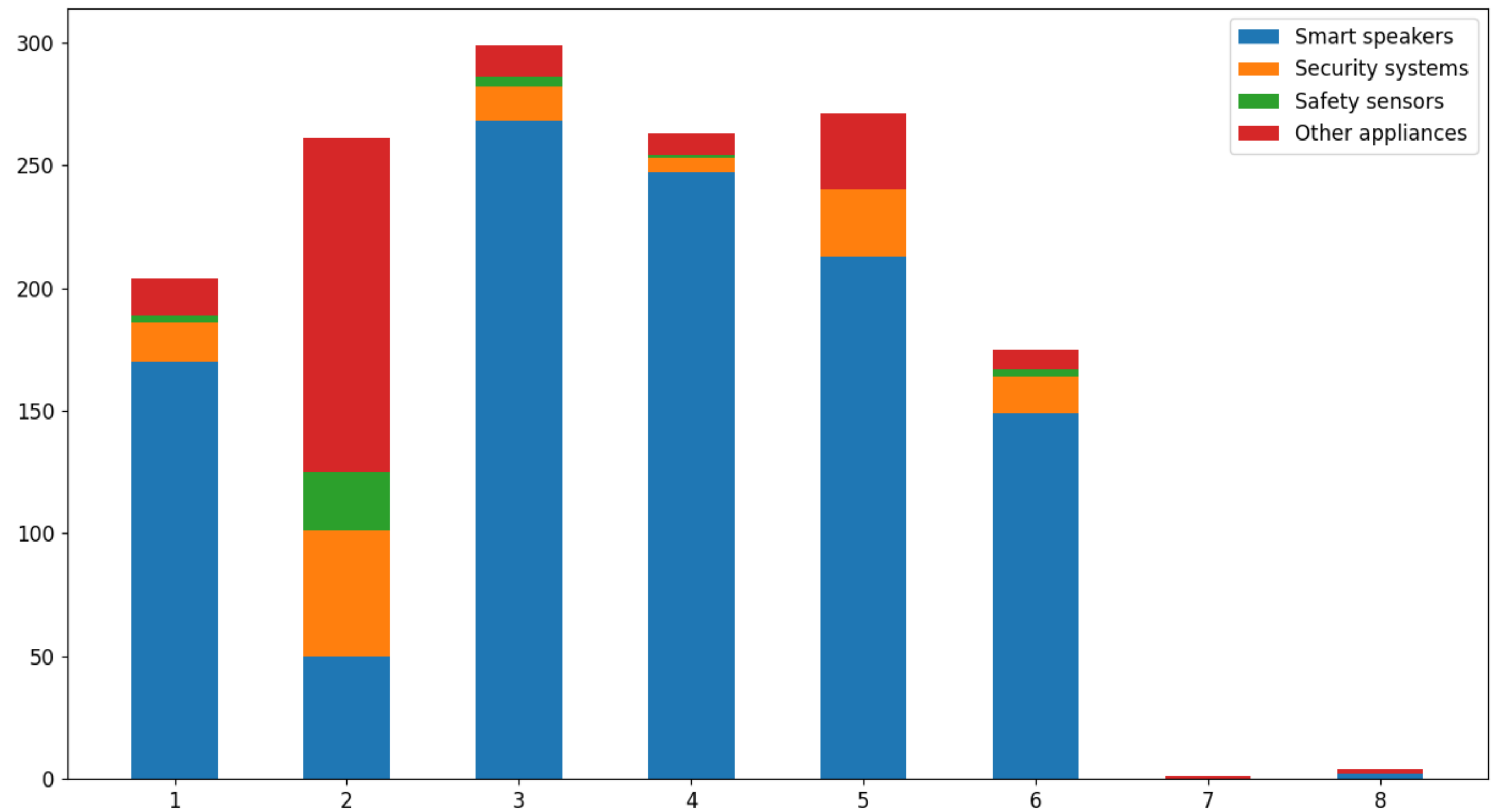

[1] I have disabled some features on my smart device (e.g. turning off the microphone or camera)

[2] I have not taken any measures to protect my privacy

[3] I avoid having sensitive conversations around my smart device

[4] I unplug my smart device when it is not in use

[5] I have limited my privacy settings on my smart device

[6] I update my smart device software regularly

[7] Other

[8] Prefer not to answer

Figure S13

- I have disabled some features on my smart device (e.g. turning off the microphone or camera) : {'Smart speakers': 170, 'Security systems': 16, 'Safety sensors': 3, 'Other appliances': 15}
- I have not taken any measures to protect my privacy : {'Smart speakers': 50, 'Security systems': 51, 'Safety sensors': 24, 'Other appliances': 136}
- I avoid having sensitive conversations around my smart device : {'Smart speakers': 268, 'Security systems': 14, 'Safety sensors': 4, 'Other appliances': 13}
- I unplug my smart device when it is not in use : {'Smart speakers': 247, 'Security systems': 6, 'Safety sensors': 1, 'Other appliances': 9}
- I have limited my privacy settings on my smart device : {'Smart speakers': 213, 'Security systems': 27, 'Other appliances': 31}
- I update my smart device software regularly : {'Smart speakers': 149, 'Security systems': 15, 'Safety sensors': 3, 'Other appliances': 8}
- Other : {'Other appliances': 1}
- Prefer not to answer : {'Smart speakers': 2, 'Other appliances': 2}

Other:

- i dont allow it to map my floor plan.

Q: Do you believe these measures will fully protect your privacy?

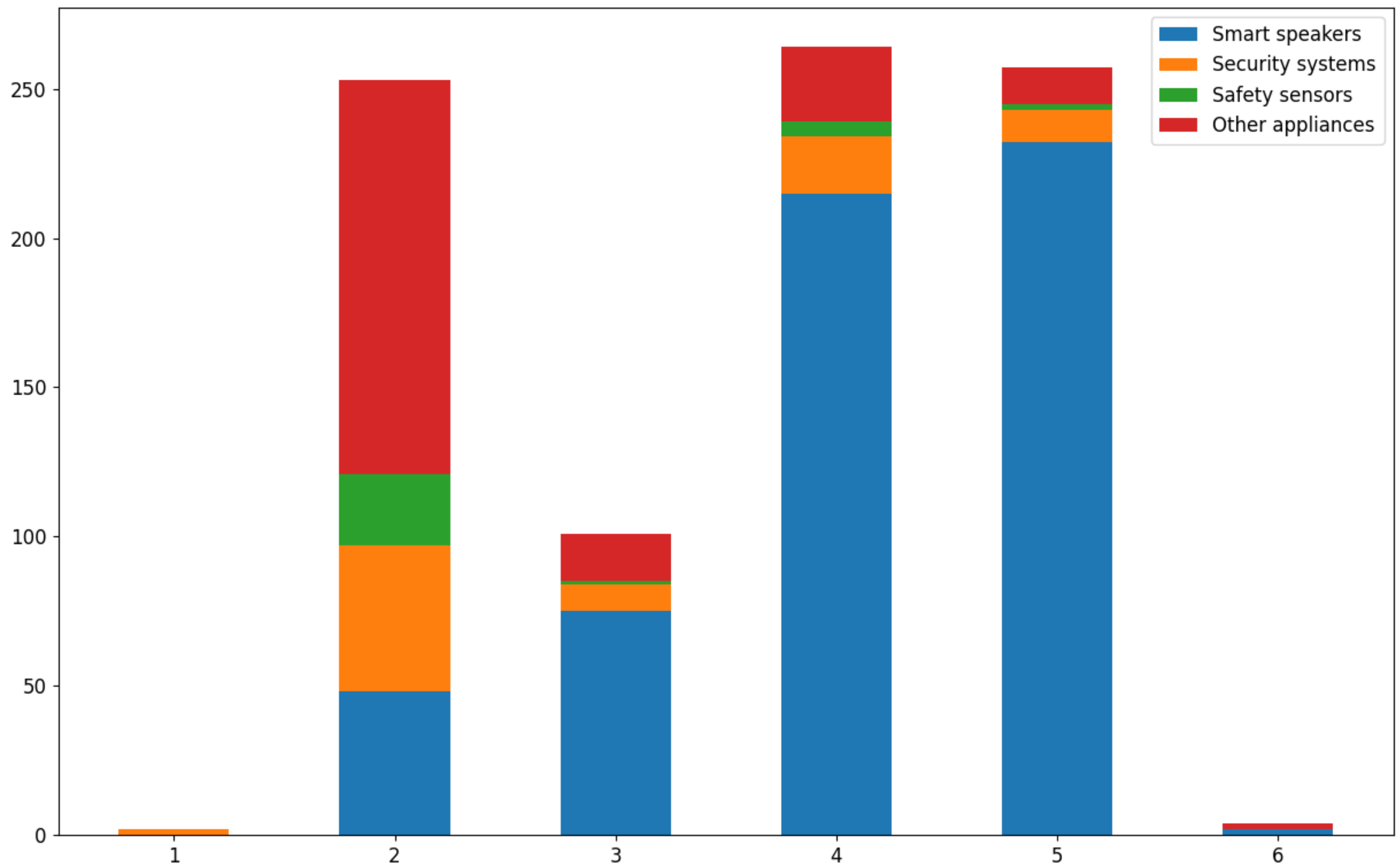

[1] I do not think these measures protect my privacy
[2] I have not taken any measures to protect my privacy
[3] I believe that these measures fully protect my privacy
[4] These measures only protect my privacy to some extent
[5] I do not know if these measures will protect my privacy, but it is out of my control
[6] No response

Figure S14

- I do not think these measures protect my privacy : {'Security systems': 2}**
- I have not taken any measures to protect my privacy : {'Smart speakers': 48, 'Security systems': 49, 'Safety sensors': 24, 'Other appliances': 132}**
- I believe that these measures fully protect my privacy : {'Smart speakers': 75, 'Security systems': 9, 'Safety sensors': 1, 'Other appliances': 16}
- These measures only protect my privacy to some extent : {'Smart speakers': 215, 'Security systems': 19, 'Safety sensors': 5, 'Other appliances': 25}
- I do not know if these measures will protect my privacy, but it is out of my control : {'Smart speakers': 232, 'Security systems': 11, 'Safety sensors': 2, 'Other appliances': 12}***
- No response : {'Smart speakers': 2, 'Other appliances': 2}

** There is some exclusion between these answers. The second answer was meant to give an option to users who don't think about their privacy at all,

***This answer was meant to address digital resignation for users who are resignedly indifferent about their privacy.

Q: Do you believe you sacrifice your privacy for the convenience of your smart object?

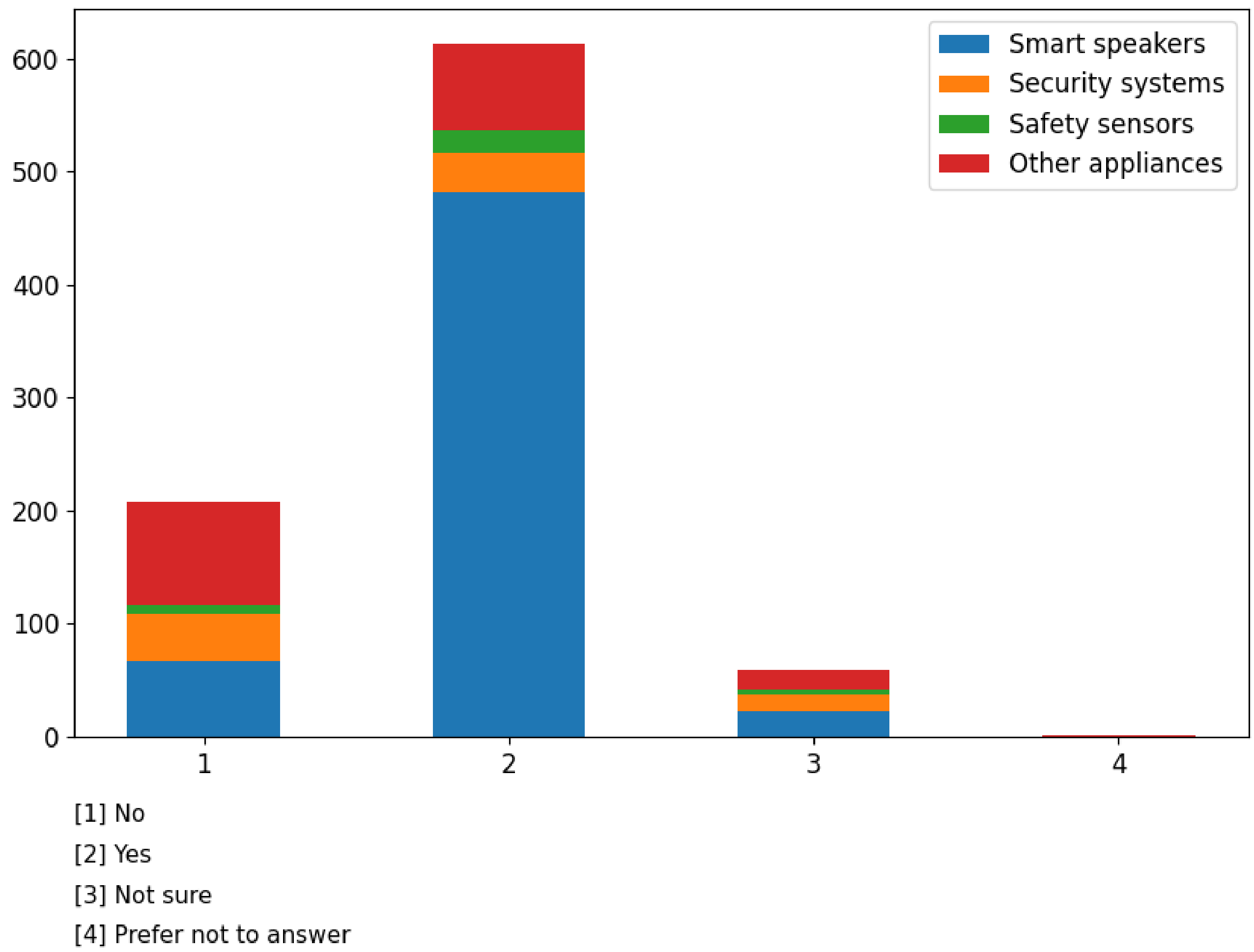

Figure S15

- No : {'Smart speakers': 67, 'Security systems': 42, 'Safety sensors': 7, 'Other appliances': 92}
- Yes : {'Smart speakers': 482, 'Security systems': 34, 'Safety sensors': 21, 'Other appliances': 76}
- Not sure : {'Smart speakers': 23, 'Security systems': 14, 'Safety sensors': 4, 'Other appliances': 18}

- Prefer not to answer : {'Other appliances': 1}

Section 4: Purpose

Q: Do you believe your smart object collects personal information from you?

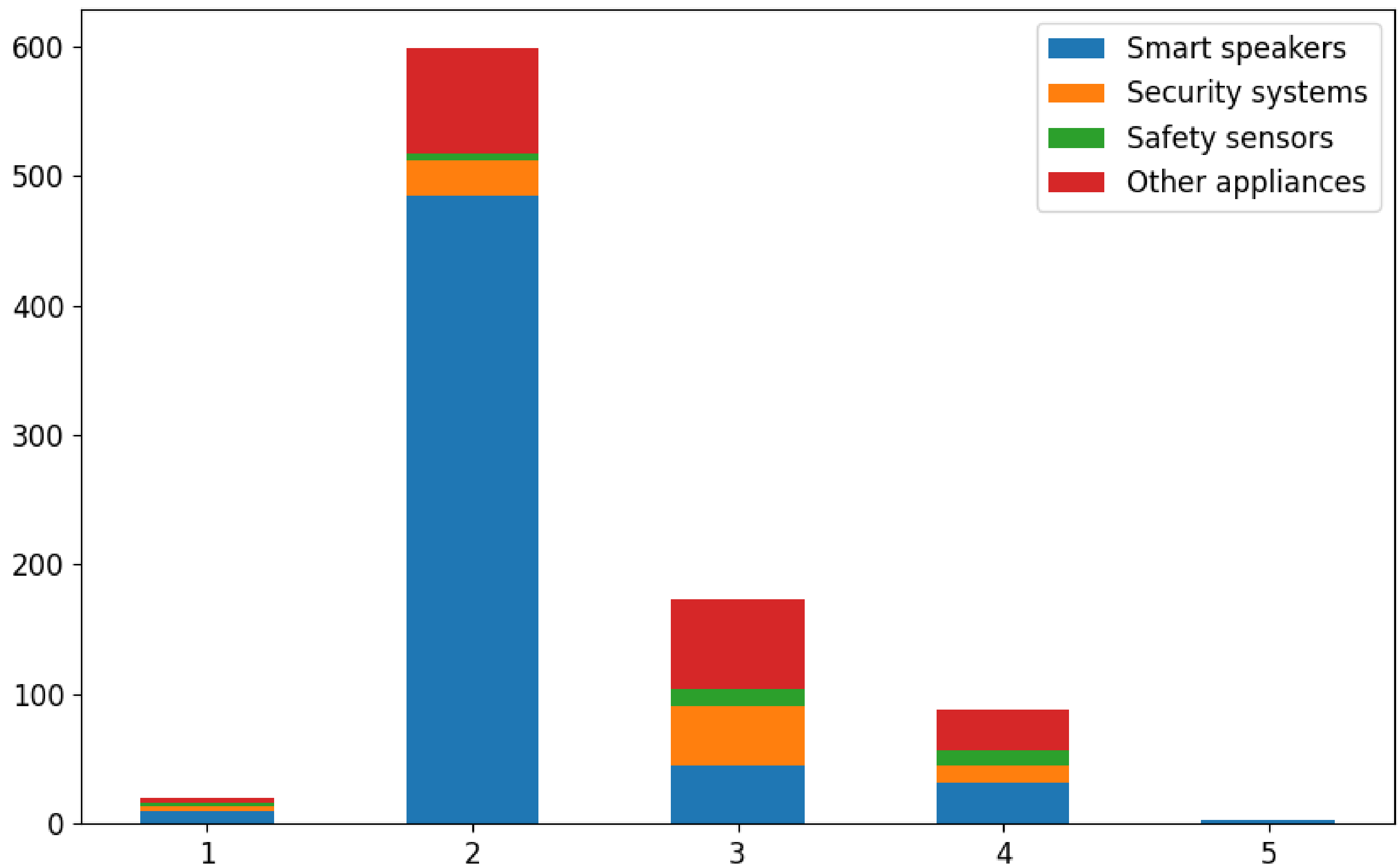

[1] My smart device constantly collects my information/monitors me
[2] My smart device does not collect any personal information
[3] My smart device collects some information, but only when I actively use it
[4] My smart device regularly collects my information, but I am not constantly monitored
[5] Prefer not to answer

Figure S16

- My smart device constantly collects my information/monitors me : {'Smart speakers': 9, 'Security systems': 4, 'Safety sensors': 3, 'Other appliances': 3}
- My smart device does not collect any personal information : {'Smart speakers': 485, 'Security systems': 27, 'Safety sensors': 5, 'Other appliances': 82}
- My smart device collects some information, but only when I actively use it : {'Smart speakers': 45, 'Security systems': 46, 'Safety sensors': 12, 'Other appliances': 70}
- My smart device regularly collects my information, but I am not constantly monitored : {'Smart speakers': 31, 'Security systems': 13, 'Safety sensors': 12, 'Other appliances': 32}
- Prefer not to answer : {'Smart speakers': 2}

Q: What kind of information do you think is collected? Select all that apply.

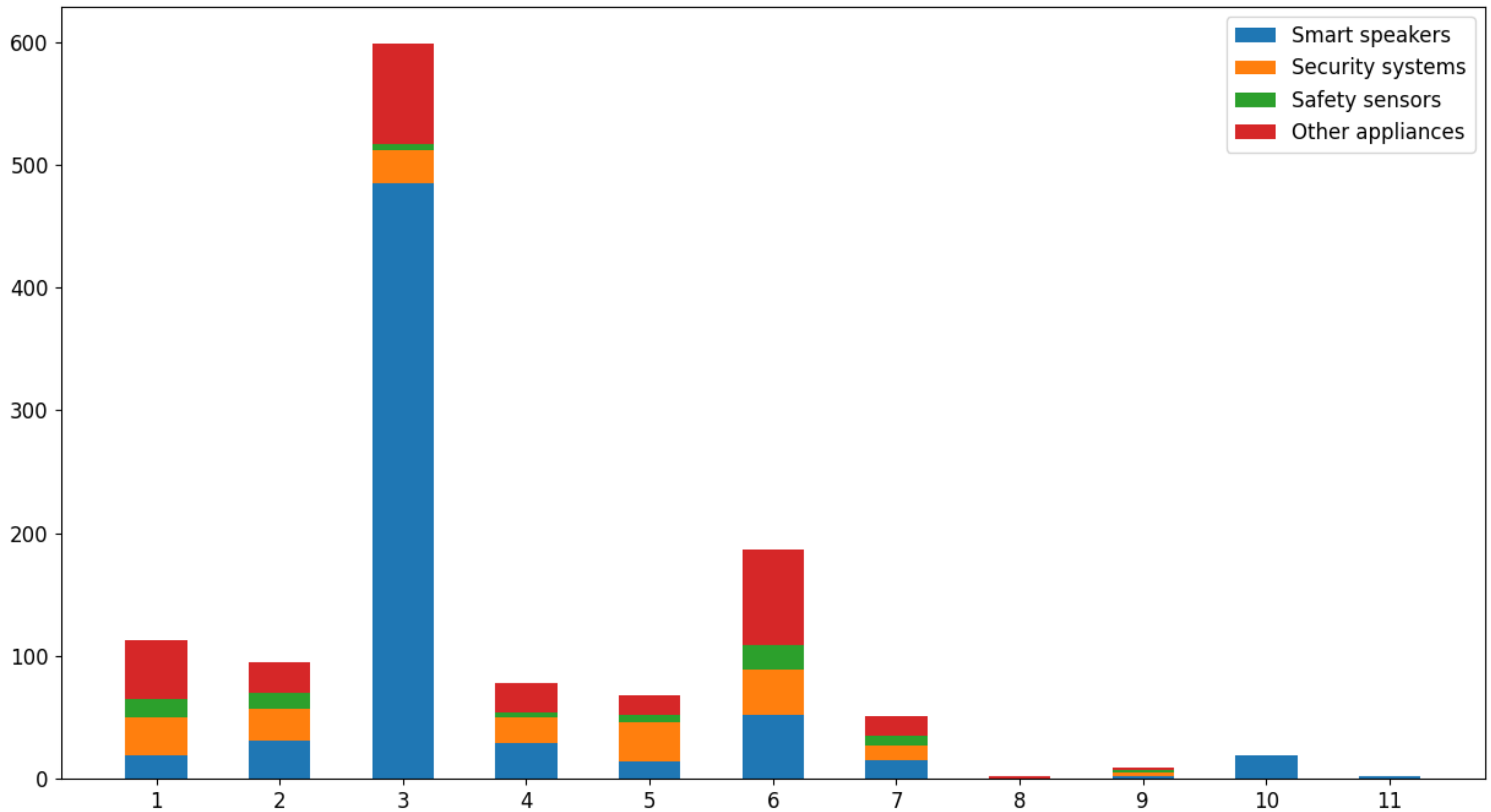

[1] Location
[2] Voice recordings
[3] I believe my smart device does not collect any personal information
[4] Search history
[5] Video recordings
[6] Timing of use of my smart device
[7] Motion (your movement within your home)
[8] Other
[9] Not sure
[10] No response
[11] Prefer not to answer

Figure S17

- Location : {'Smart speakers': 19, 'Security systems': 31, 'Safety sensors': 15, 'Other appliances': 48}
- Voice recordings : {'Smart speakers': 31, 'Security systems': 26, 'Safety sensors': 13, 'Other appliances': 25}
- I believe my smart device does not collect any personal information : {'Smart speakers': 485, 'Security systems': 27, 'Safety sensors': 5, 'Other appliances': 82}
- Search history : {'Smart speakers': 29, 'Security systems': 21, 'Safety sensors': 4, 'Other appliances': 24}
- Video recordings : {'Smart speakers': 14, 'Security systems': 32, 'Safety sensors': 6, 'Other appliances': 16}
- Timing of use of my smart device : {'Smart speakers': 52, 'Security systems': 37, 'Safety sensors': 20, 'Other appliances': 78}
- Motion (your movement within your home) : {'Smart speakers': 15, 'Security systems': 12, 'Safety sensors': 8, 'Other appliances': 16}
- Other : {'Other appliances': 2}
- Not sure : {'Smart speakers': 2, 'Security systems': 3, 'Safety sensors': 2, 'Other appliances': 2}
- No response : {'Smart speakers': 19}
- Prefer not to answer : {'Smart speakers': 2}

Other:
- layout of my home
- Sleep time

Q: Why do you believe your personal information is collected? Select all that apply.

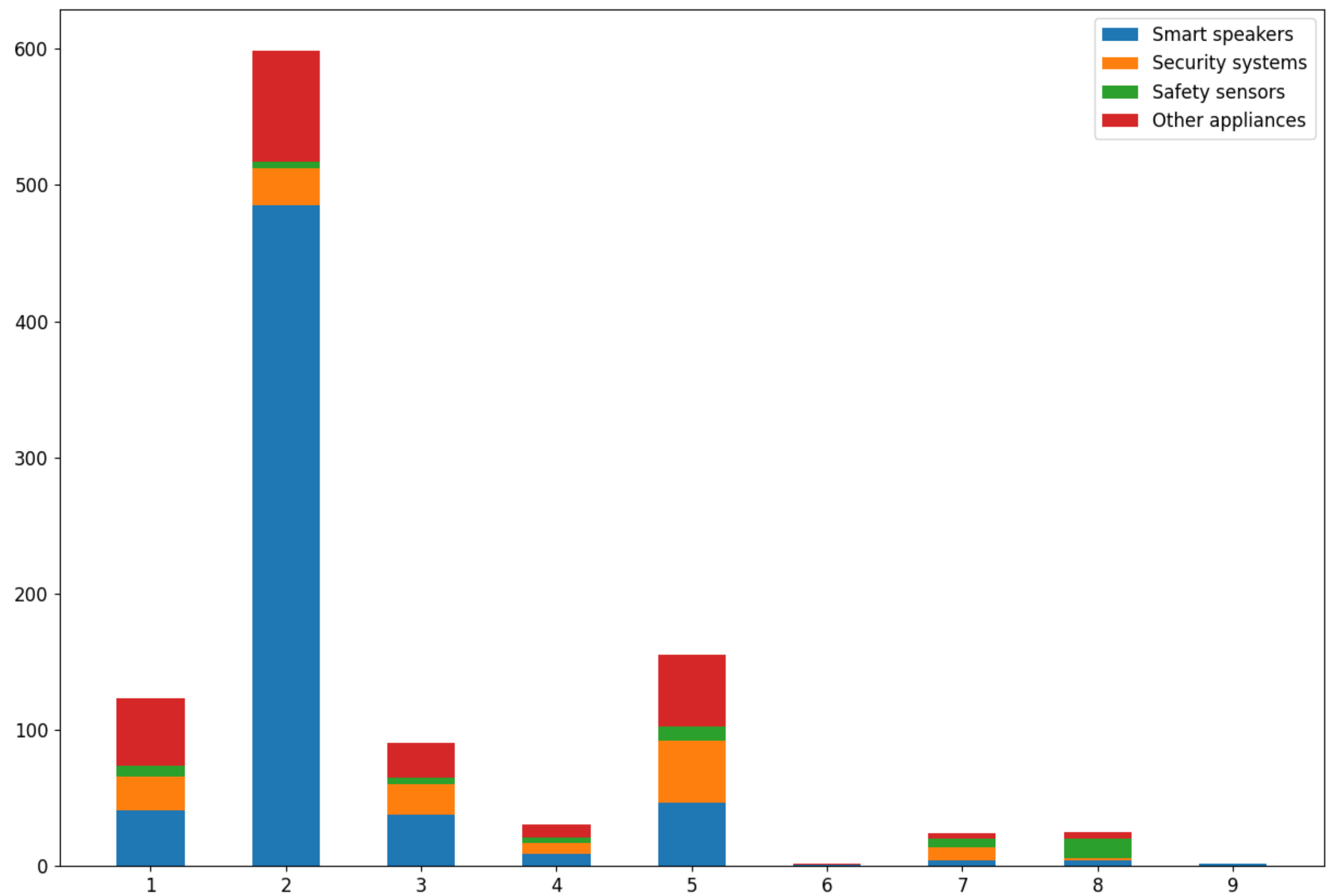

[1] My smart device collects my information both for its function and for the smart device producers (i.e. companies) to store and use for marketing purposes

[2] I believe my smart device does not collect any personal information

[3] My smart device likely collects personal information that is unnecessary for its function, but I do not know why

[4] My smart device collects my information so the government can monitor me

[5] My smart device collects my information, but it is essential for its function

[6] Not sure

[7] My smart device collects my information for healthcare reasons

[8] My smart device collects my information so my family can monitor me

[9] Prefer not to answer

Figure S18

- My smart device collects my information both for its function and for the smart device producers (i.e. companies) to store and use for marketing purposes : {'Smart speakers': 41, 'Security systems': 25, 'Safety sensors': 8, 'Other appliances': 49}
- I believe my smart device does not collect any personal information : {'Smart speakers': 485, 'Security systems': 27, 'Safety sensors': 5, 'Other appliances': 82}
- My smart device likely collects personal information that is unnecessary for its function, but I do not know why : {'Smart speakers': 38, 'Security systems': 22, 'Safety sensors': 5, 'Other appliances': 26}**
- My smart device collects my information so the government can monitor me : {'Smart speakers': 9, 'Security systems': 8, 'Safety sensors': 4, 'Other appliances': 10}
- My smart device collects my information, but it is essential for its function : {'Smart speakers': 47, 'Security systems': 45, 'Safety sensors': 11, 'Other appliances': 52}
- Not sure : {'Smart speakers': 1, 'Other appliances': 1}
- My smart device collects my information for healthcare reasons : {'Smart speakers': 4, 'Security systems': 10, 'Safety sensors': 6, 'Other appliances': 4}
- My smart device collects my information so my family can monitor me : {'Smart speakers': 4, 'Security systems': 2, 'Safety sensors': 14, 'Other appliances': 5}
- Prefer not to answer : {'Smart speakers': 2}

** users can have *in*complete rather than ignorant understandings of the data collection process—that's how some users could know that unnecessary data collection occurs without knowing why exactly a company would bother collecting unnecessary data.

Q: Have you read the Terms and Conditions for your smart object in detail?

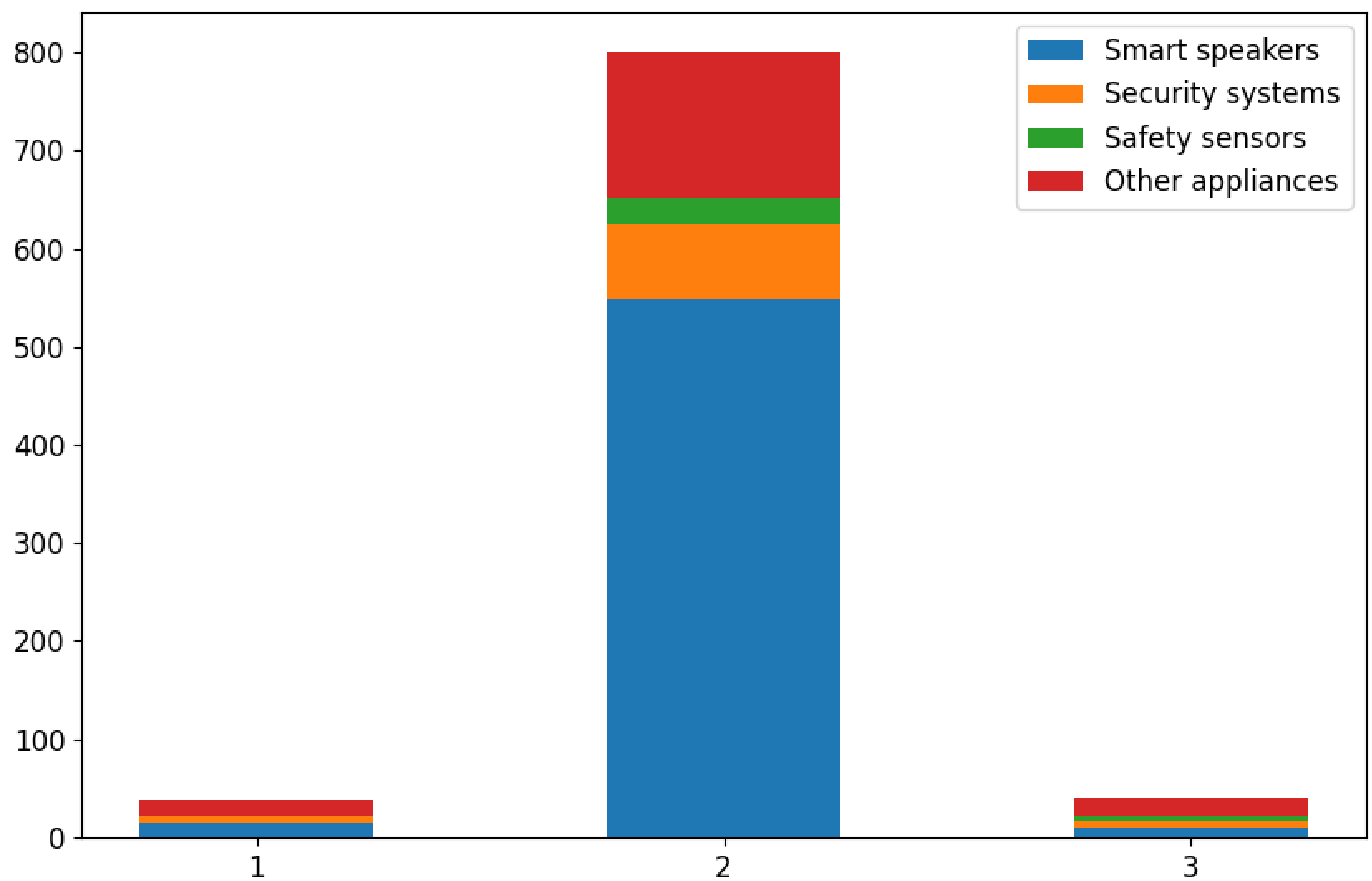

[1] No

[2] Yes

[3] Not sure

Figure S19

- No : {'Smart speakers': 15, 'Security systems': 6, 'Safety sensors': 1, 'Other appliances': 17}
- Yes : {'Smart speakers': 548, 'Security systems': 76, 'Safety sensors': 27, 'Other appliances': 150}
- Not sure : {'Smart speakers': 9, 'Security systems': 8, 'Safety sensors': 4, 'Other appliances': 20}

Section 5: Risk

Q: When you received your smart object, were you concerned about any risks? These risks could include privacy loss, malfunction, or just hassle.

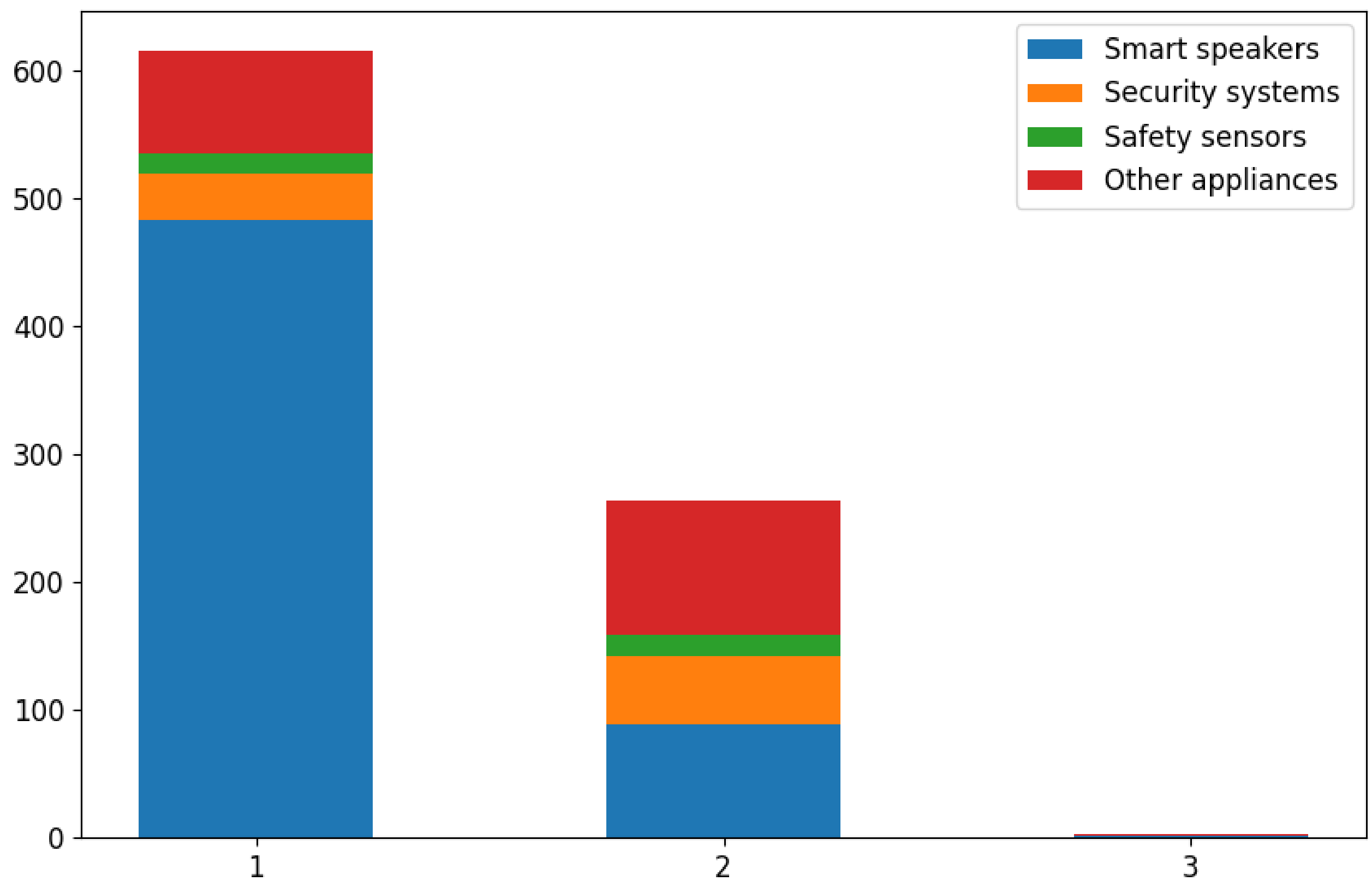

[1] Yes

[2] No

[3] Prefer not to answer

Figure S20

- Yes : {'Smart speakers': 483, 'Security systems': 37, 'Safety sensors': 15, 'Other appliances': 81}
- No : {'Smart speakers': 88, 'Security systems': 53, 'Safety sensors': 17, 'Other appliances': 105}
- Prefer not to answer : {'Smart speakers': 1, 'Other appliances': 1}

Q: Which potential risks were you concerned about? Select all that apply.

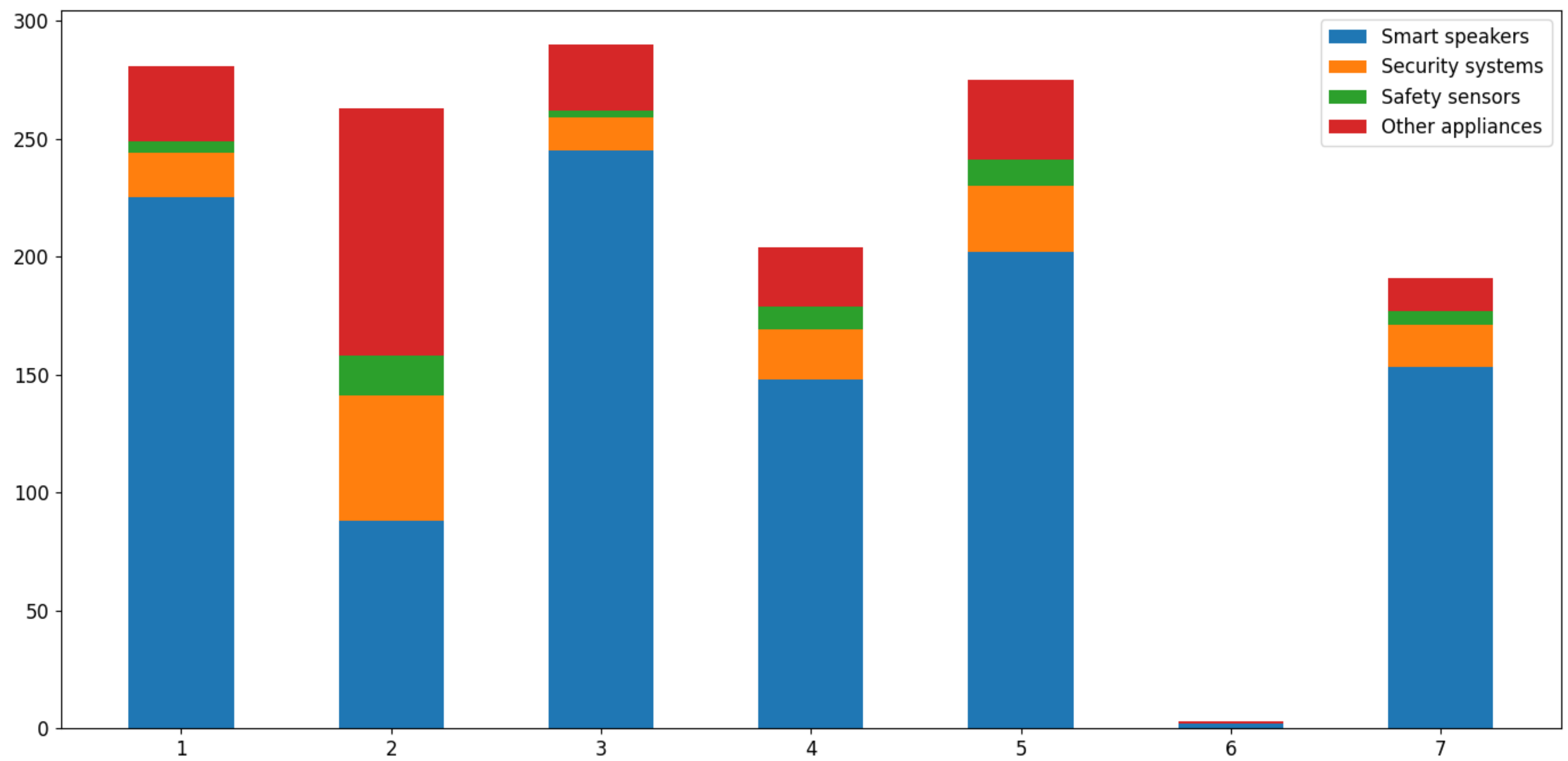

[1] I was concerned with losing my personal information

[2] I am not concerned about risks.

[3] I was concerned I could be monitored

[4] I was concerned my smart device would underperform

[5] I was concerned my smart device would malfunction

[6] Prefer not to answer

[7] I was concerned that my smart device could be hacked

Figure S21

- I was concerned with losing my personal information : {'Smart speakers': 225, 'Security systems': 19, 'Safety sensors': 5, 'Other appliances': 32}
- I am not concerned about risks. : {'Smart speakers': 88, 'Security systems': 53, 'Safety sensors': 17, 'Other appliances': 105}
- I was concerned I could be monitored : {'Smart speakers': 245, 'Security systems': 14, 'Safety sensors': 3, 'Other appliances': 28}
- I was concerned my smart device would underperform : {'Smart speakers': 148, 'Security systems': 21, 'Safety sensors': 10, 'Other appliances': 25}
- I was concerned my smart device would malfunction : {'Smart speakers': 202, 'Security systems': 28, 'Safety sensors': 11, 'Other appliances': 34}
- Prefer not to answer : {'Smart speakers': 2, 'Other appliances': 1}
- I was concerned that my smart device could be hacked : {'Smart speakers': 153, 'Security systems': 18, 'Safety sensors': 6, 'Other appliances': 14}

Q: Do you think that the risks could be reduced by new policies, new laws, or by the device designers?

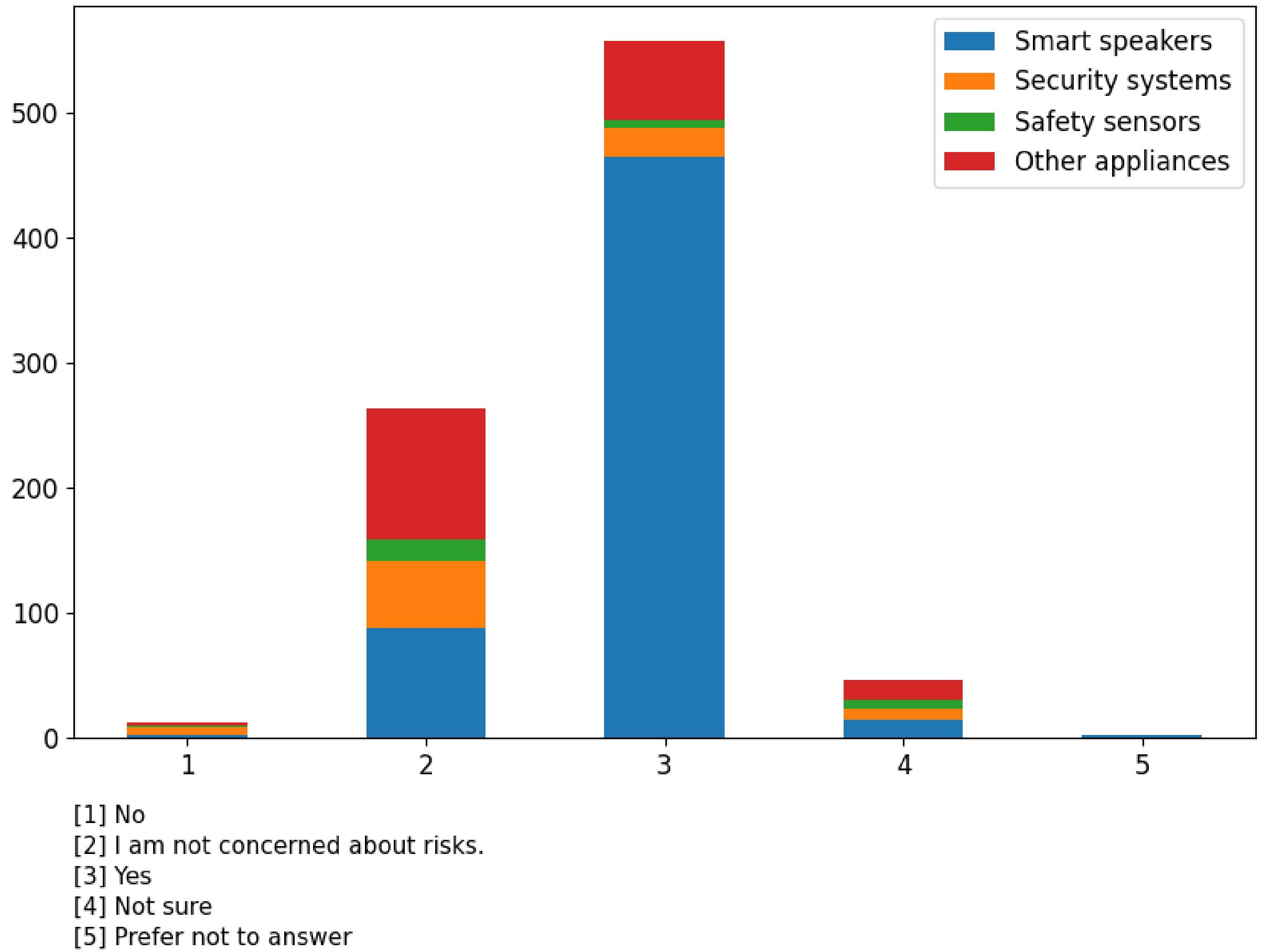

Figure S22

- No : {'Smart speakers': 3, 'Security systems': 6, 'Safety sensors': 1, 'Other appliances': 2}
- I am not concerned about risks. : {'Smart speakers': 88, 'Security systems': 53, 'Safety sensors': 17, 'Other appliances': 105}

- Yes : {'Smart speakers': 464, 'Security systems': 23, 'Safety sensors': 7, 'Other appliances': 63}
- Not sure : {'Smart speakers': 15, 'Security systems': 8, 'Safety sensors': 7, 'Other appliances': 16}
- Prefer not to answer : {'Smart speakers': 2, 'Other appliances': 1}

Q: Have your perceptions of these risks changed from when you received your smart object until now?

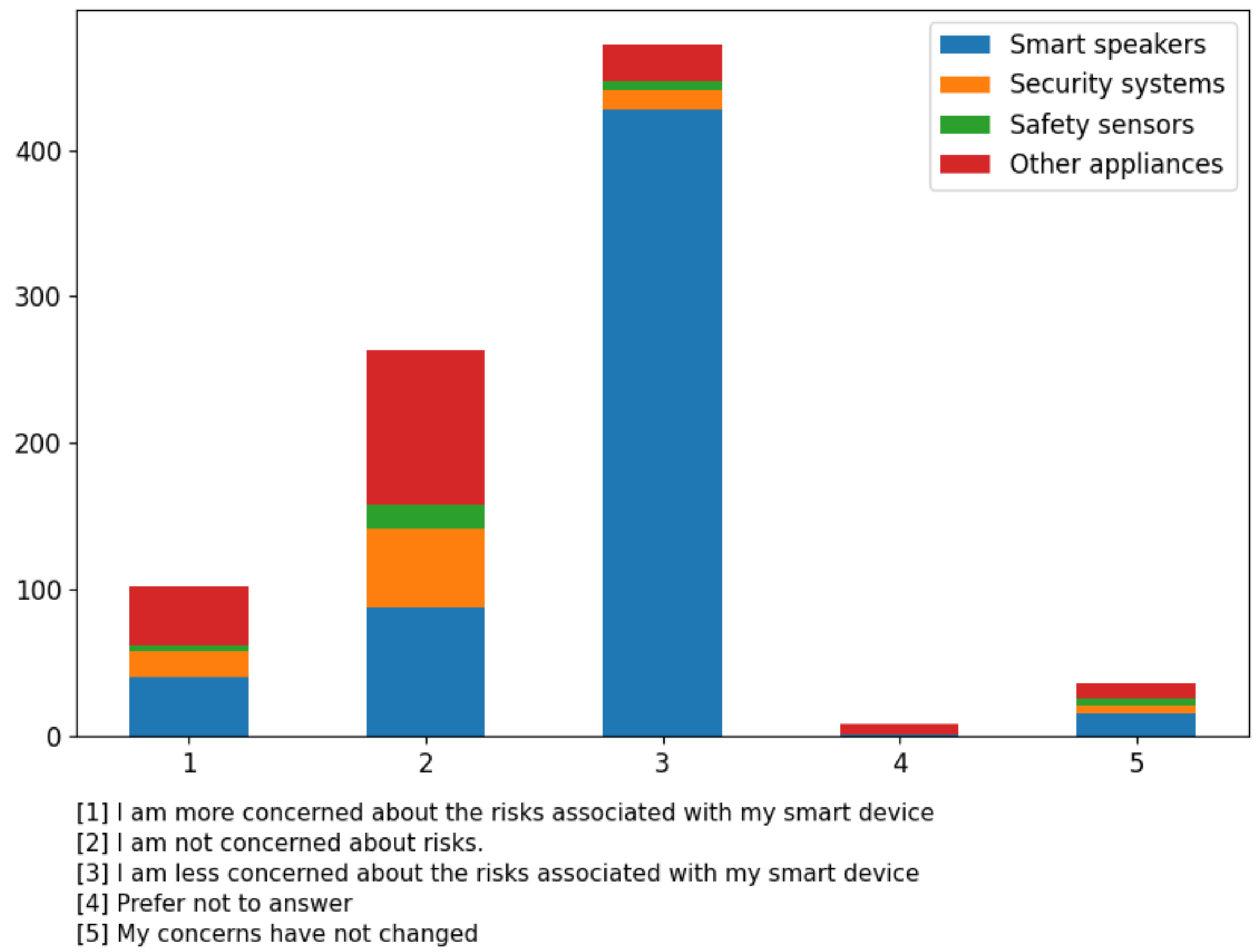

Figure S23

- I am more concerned about the risks associated with my smart device : {'Smart speakers': 40, 'Security systems': 18, 'Safety sensors': 4, 'Other appliances': 40}
- I am not concerned about risks. : {'Smart speakers': 88, 'Security systems': 53, 'Safety sensors': 17, 'Other appliances': 105}
- I am less concerned about the risks associated with my smart device : {'Smart speakers': 427, 'Security systems': 14, 'Safety sensors': 6, 'Other appliances': 25}

- Prefer not to answer : {'Smart speakers': 1, 'Other appliances': 7}
- My concerns have not changed : {'Smart speakers': 16, 'Security systems': 5, 'Safety sensors': 5, 'Other appliances': 10}

Q: What are the benefits of your smart object, if any? Select all that apply.

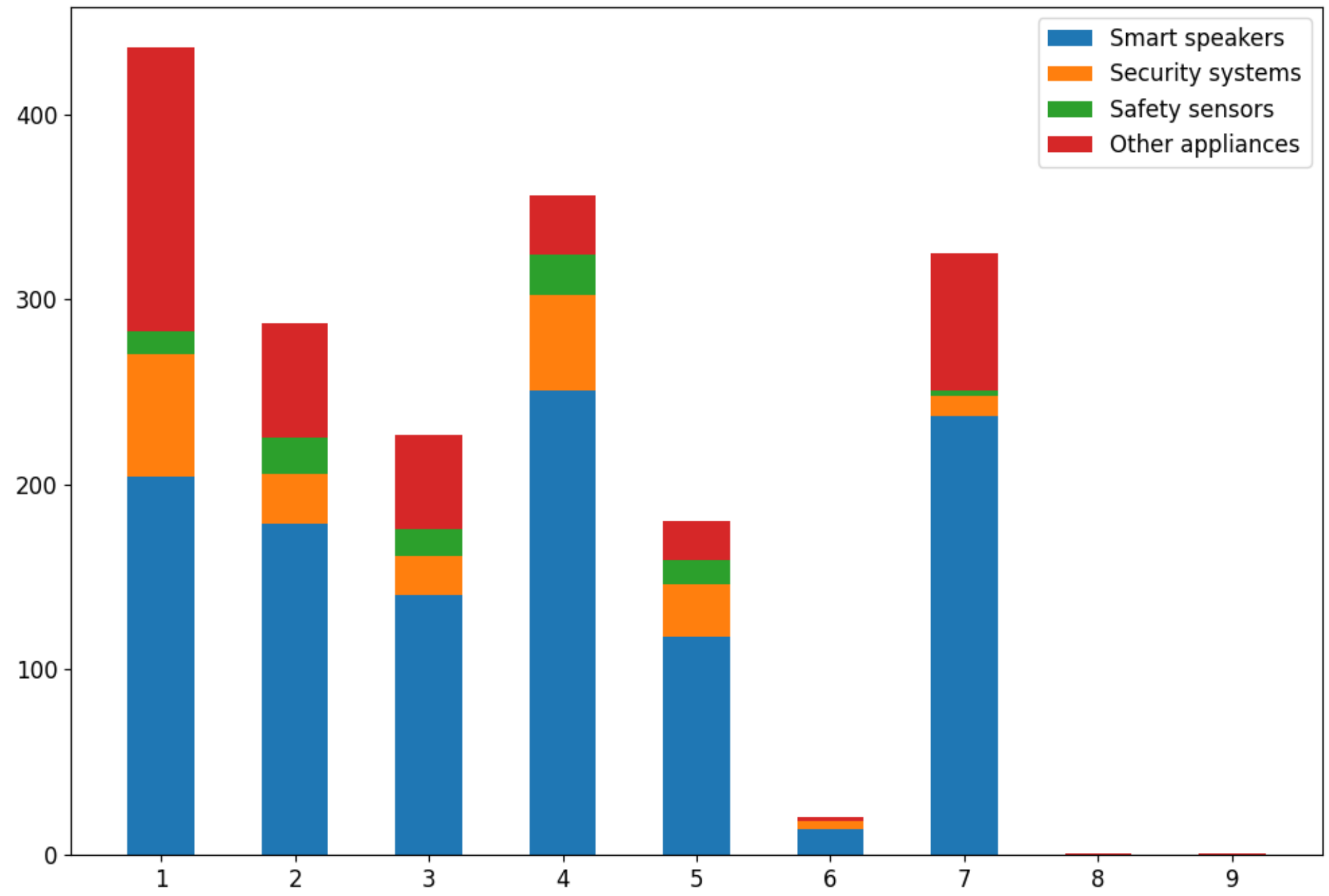

[1] Enhanced daily convenience
[2] Health benefits
[3] Increased independence
[4] Enhanced safety
[5] Enhanced security
[6] No benefits
[7] Low cost
[8] Prefer not to answer
[9] Other

Figure S24

- Enhanced daily convenience : {'Smart speakers': 204, 'Security systems': 66, 'Safety sensors': 13, 'Other appliances': 153}
- Health benefits : {'Smart speakers': 179, 'Security systems': 27, 'Safety sensors': 19, 'Other appliances': 62}
- Increased independence : {'Smart speakers': 140, 'Security systems': 21, 'Safety sensors': 15, 'Other appliances': 51}
- Enhanced safety : {'Smart speakers': 251, 'Security systems': 51, 'Safety sensors': 22, 'Other appliances': 32}
- Enhanced security : {'Smart speakers': 118, 'Security systems': 28, 'Safety sensors': 13, 'Other appliances': 21}
- No benefits : {'Smart speakers': 14, 'Security systems': 4, 'Other appliances': 2}
- Low cost : {'Smart speakers': 237, 'Security systems': 11, 'Safety sensors': 3, 'Other appliances': 74}
- Prefer not to answer : {'Other appliances': 1}
- Other : {'Other appliances': 1}

Other:

- Entertainment

Q: Did the benefits of your smart object outweigh the concerns your selected above?

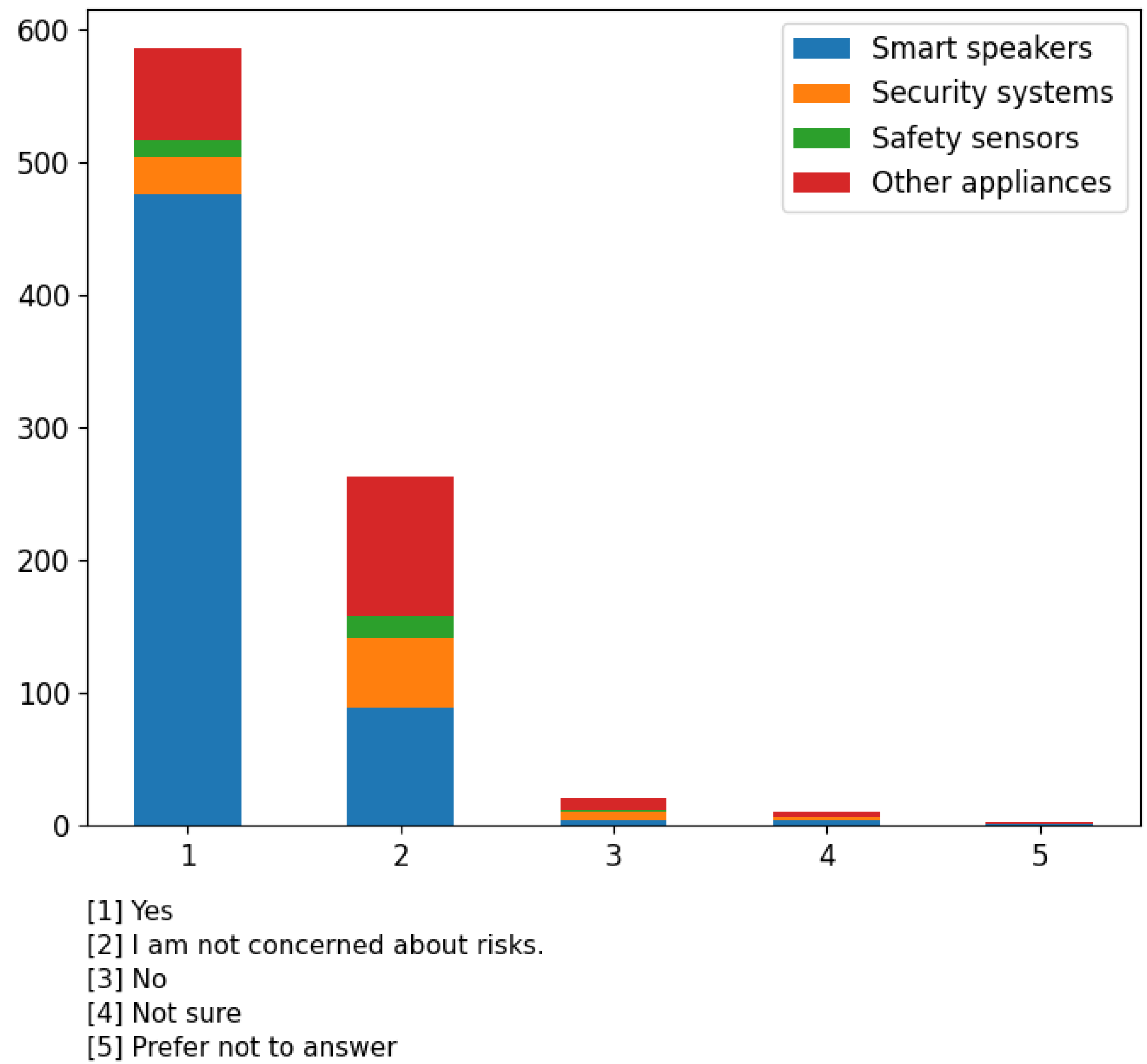

Figure S25

- Yes : {'Smart speakers': 475, 'Security systems': 29, 'Safety sensors': 13, 'Other appliances': 69}
- I am not concerned about risks. : {'Smart speakers': 88, 'Security systems': 53, 'Safety sensors': 17, 'Other appliances': 105}

- No : {'Smart speakers': 4, 'Security systems': 6, 'Safety sensors': 1, 'Other appliances': 9}
- Not sure : {'Smart speakers': 4, 'Security systems': 2, 'Safety sensors': 1, 'Other appliances': 3}
- Prefer not to answer : {'Smart speakers': 1, 'Other appliances': 1}

Section 6: Safety

Q: Does your smart object make you feel safer in your home?

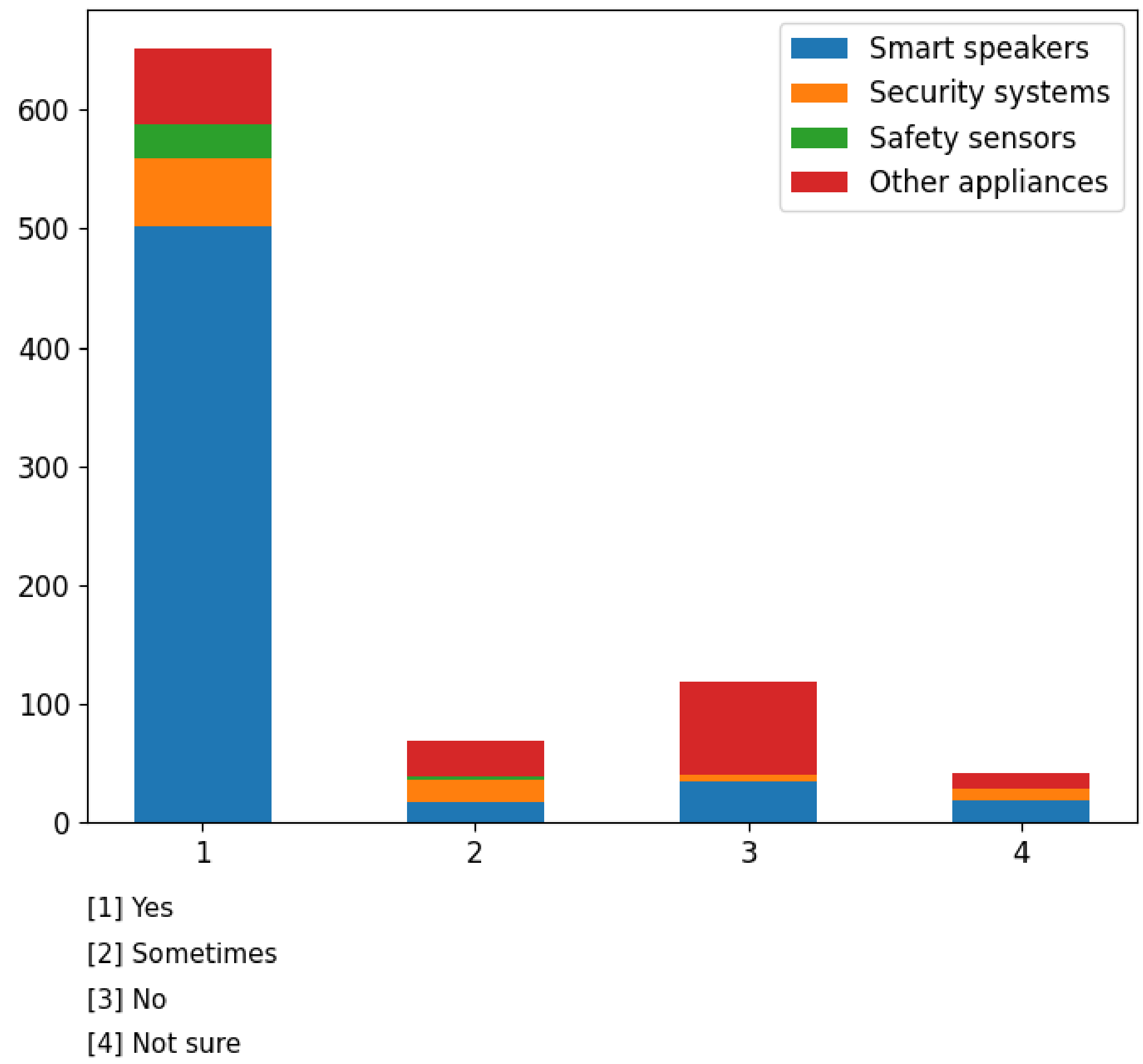

Figure S26

- Yes : {'Smart speakers': 502, 'Security systems': 57, 'Safety sensors': 28, 'Other appliances': 65}

- Sometimes : {'Smart speakers': 17, 'Security systems': 18, 'Safety sensors': 4, 'Other appliances': 30}
- No : {'Smart speakers': 34, 'Security systems': 6, 'Other appliances': 78}
- Not sure : {'Smart speakers': 19, 'Security systems': 9, 'Other appliances': 14}

Q: Why you feel safer? Select all that apply.

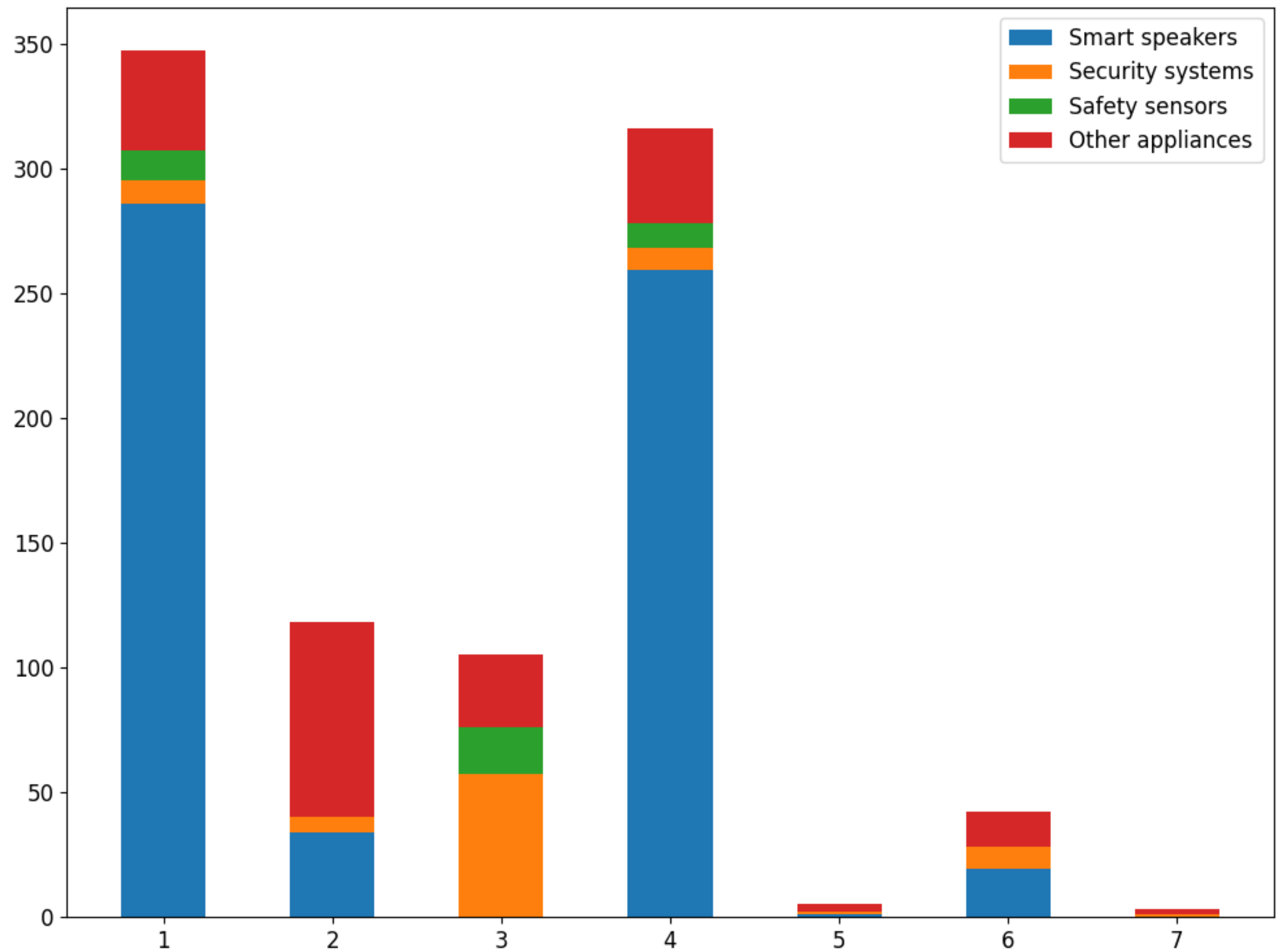

[1] My smart device could detect and connect me to emergency services quickly if I am in danger (e.g., fall, heart attack, violence)
[2] I dont feel safer.
[3] No response
[4] I could connect with family (or vice versa) more easily during an emergency
[5] Prefer not to answer
[6] Not sure
[7] Other

Figure S27

- My smart device could detect and connect me to emergency services quickly if I am in danger (e.g., fall, heart attack, violence) : {'Smart speakers': 286, 'Security systems': 9, 'Safety sensors': 12, 'Other appliances': 40}
- I dont feel safer. : {'Smart speakers': 34, 'Security systems': 6, 'Other appliances': 78}
- No response : {'Security systems': 57, 'Safety sensors': 19, 'Other appliances': 29}
- I could connect with family (or vice versa) more easily during an emergency : {'Smart speakers': 259, 'Security systems': 9, 'Safety sensors': 10, 'Other appliances': 38}
- Prefer not to answer : {'Smart speakers': 1, 'Security systems': 1, 'Other appliances': 3}
- Not sure : {'Smart speakers': 19, 'Security systems': 9, 'Other appliances': 14}
- Other : {'Security systems': 1, 'Other appliances': 2}

Other:

- Convenient and quick
- Control over home environment
- We have recorded evidence of any activity by those who should not be on our property
